# qNEP: A Highly Efficient Neuroevolution Potential with Dynamic Charges for Large-Scale Atomistic Simulations

**DOI:** 10.1021/acs.jctc.6c00146

**Published:** 2026-04-20

**Authors:** Zheyong Fan, Benrui Tang, Esmée Berger, Ethan Berger, Erik Fransson, Ke Xu, Zihan Yan, Zhoulin Liu, Zichen Song, Haikuan Dong, Shunda Chen, Lei Li, Ziliang Wang, Yizhou Zhu, Julia Wiktor, Paul Erhart

**Affiliations:** † College of Physical Science and Technology, 12687 Bohai University, Jinzhou 121013, P. R. China; ‡ Suzhou Laboratory, Suzhou, Jiangsu 215123, P. R. China; § Department of Physics, 11248 Chalmers University of Technology, 41296 Gothenburg, Sweden; ∥ Department of Materials Science and Engineering, 557712 Westlake University, Hangzhou, Zhejiang 310030, P. R. China; ⊥ School of Science, 529484 Harbin Institute of Technology, Shenzhen, Guangdong 518055, P. R. China; # Shenzhen Key Laboratory of Micro/Nano-Porous Functional Materials (SKLPM), Department of Materials Science and Engineering, 255310 Southern University of Science and Technology, Shenzhen 518055, P. R. China; ∇ Department of Materials Science and Engineering, City University of Hong Kong, Hong Kong SAR 999077, P. R. China; ○ Department of Civil and Environmental Engineering, 8367 George Washington University, Washington, District of Columbia 20052, United States; ◆ National Engineering Laboratory for Reducing Emissions from Coal Combustion, Shandong Key Laboratory of Green Thermal Power and Carbon Reduction, 201400 Shandong University, Jinan, Shandong 250061, P. R. China; ¶ Wallenberg Initiative Materials Science for Sustainability, Chalmers University of Technology, 41926 Gothenburg, Sweden

## Abstract

Although electrostatics can be incorporated into machine-learned interatomic potentials, existing approaches are computationally very demanding, limiting large-scale, long-time simulations of electrostatics-driven phenomena such as dielectric response, infrared activity, and field–matter coupling. Here, we extend the neuroevolution potential (NEP), a highly efficient machine-learned interatomic potential, to a charge-aware framework (qNEP) by introducing explicit, environment-dependent partial charges. Each ionic partial charge is represented by a neural network as a function of the local descriptor vector, analogous to the NEP site-energy model. This formulation enables the direct prediction of the Born effective charge tensor for each ion and, consequently, the polarization. As a result, dielectric properties, infrared spectra, and coupling to external electric fields can be evaluated within a unified framework. We derive consistent expressions for the forces and virials that explicitly account for the position dependence of the partial charges. The qNEP method has been implemented in the free-and-open-source GPUMD package with support for both Ewald summation and particle–particle particle–mesh treatments of electrostatics. We demonstrate the accuracy and efficiency of the qNEP approach through representative applications to water, Li_7_La_3_Zr_2_O_12_, BaTiO_3_, and a magnesium–water interface. These results show that qNEP enables accurate atomistic simulations with explicit long-range electrostatics, scalable to million-atom systems on nanosecond time scales using consumer-grade GPUs.

## Graphical abstract

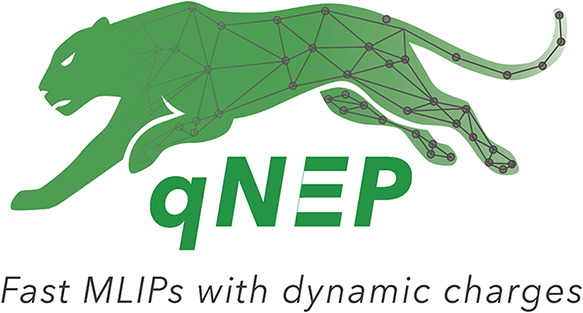

## 1 Introduction

Machine-learned interatomic potentials (MLIPs) have become a widely adopted approach for accurate and efficient atomic-scale modeling of materials. Early MLIPs^1,2^ were inherently short-ranged. This approximation is adequate for many systems because of the short-sightedness of chemical bonding. However, short-ranged models become inadequate in systems with sizable partial charges and weak screening, where electrostatic interactions are intrinsically long-ranged. They are also limited when explicit coupling to external electric fields is required.

A common strategy to incorporate long-range electrostatics is to introduce fixed charges and subtract electrostatic contributions to energy and forces from the reference data.^2–4^ A more flexible alternative employs a separate regression model, such as a neural network (NN), to predict partial charges, as in third-generation high-dimensional neural network potentials.^5,6^ In this framework, partial charges are fitted to reference values obtained from a static charge decomposition scheme. Such an approach is conceptually unsatisfactory because there is no unique decomposition of the electronic charge density into individual ionic contributions. Other methods avoid explicit ionic charge partitioning by targeting higher-order electrostatic observables, such as the dipole moment,^7^ or by representing long-range electrostatics using the centers of maximally localized Wannier functions.^8,9^

More recently, charge equilibration schemes originally developed for conventional interatomic potentials^10,11^ have been adapted for use with MLIPs.^12,13^ In contrast to approaches in which partial charges are predicted directly by a regression model, charge equilibration schemes determine the charges self-consistently by minimizing an electrostatic energy functional subject to global constraints. This formulation enforces charge conservation and enables a physically consistent description of long-range charge transfer. Such schemes are employed, for example, in the fourth-generation high-dimensional neural network potential,^12^ but they substantially increase the computational cost due to the expensive charge equilibration step, even when iterative solvers are used for acceleration.^14^

An alternative route to charge conservation is to start from the electric enthalpy and obtain the Born effective charges (BECs) as derivatives of the polarization. While this approach is physically elegant and internally consistent, current implementations^15^ rely on equivariant neural networks, which are computationally demanding.

To circumvent the reliance on reference charges and at least partly alleviate the need for explicit charge equilibration schemes, several approaches have been developed in which partial charges are not learned explicitly. Instead, they are treated as latent features of the model and determined implicitly by fitting the sum of the electrostatic energy and a short-range MLIP to the total target energies and forces. Song et al.^16^ treated the partial charges by including both real-space (short-ranged) and reciprocal-space (long-ranged) electrostatic contributions, whereas Cheng et al. considered only the reciprocal-space (long-ranged) component.^17,18^ The latter, so-called latent Ewald summation (LES) approach, also enables the calculation of the polarization and BECs,^19^ and is available as a PyTorch-based library.^20^ Related ideas have also been explored within a variational charge equilibration framework, which likewise enables the learning of partial charges without reference values.^21^ (A concise comparison of selected approaches for handling electrostatic interactions is provided in Table S1.)

Although the general principles for the incorporation of electrostatics into MLIPs have been established, existing approaches remain computationally demanding. As a result, their application to large-scale systems comprising hundreds of thousands to millions of atoms, as well as to long time scales extending from several to tens of nanoseconds, is severely constrained. This limits their use in studies of phenomena that critically depend on long-range electrostatics and polarization, such as ion and proton transport, charged defects and defect migration, dielectric response, vibrational or infrared spectroscopy, and field-driven polarization dynamics. Even for smaller systems, improving computational efficiency is essential to enable extensive sampling and efficient use of modern computing resources.

In the present work, we therefore develop qNEP, a charge-aware MLIP that combines physical fidelity with high computational efficiency, enabling predictive simulations across broad classes of materials and extended length and time scales. The qNEP framework builds on the neuroevolution potential (NEP) scheme, a highly efficient short-ranged MLIP architecture with demonstrated accuracy and performance across a wide range of materials and applications.^22–24^ Following earlier work,^16,17^ we treat the partial charges as latent features of the model and obtain the BECs as derivatives of the polarization. Charge conservation is already strongly encouraged during training through an explicit regularization term. This requires only a small numerical adjustment during simulations to ensure physically consistent electrostatics. This formulation enables the direct computation of dielectric properties and infrared spectra, as well as a consistent coupling to external electric fields.

By implementing the particle–particle particle–mesh (PPPM) method^25^ to evaluate electrostatic interactions during molecular dynamics MD simulations, we obtain a highly performant approach that is only 1.5–3 times slower than equivalently trained NEP models, while offering both enhanced functionality and improved accuracy. We demonstrate the accuracy and efficiency of qNEP through representative applications to water, Li_7_La_3_Zr_2_O_12_, BaTiO_3_, and a magnesium–water interface. These results show that qNEP enables accurate atomistic simulations with explicit long-range electrostatics, scalable to million-atom systems on nanosecond time scales using consumer-grade GPUs.

## 2 Methods

### 2.1 The Original NEP Model Architecture

The qNEP approach introduced below is based on the NEP framework,^26^ which has undergone several refinements in recent years.^27–29^ In this section, we provide a brief overview of the most recent version, NEP4.^29^ The term “neuroevolution” refers to the combination of a neural network (NN) model and an evolutionary training algorithm, namely, the separable natural evolution strategy (SNES).^30^

The machine-learning model used in NEP is a feed-forward NN with a single hidden layer (Figure 1a; blue output layer only). In terms of the NN model, the site energy can be explicitly expressed as

1 $$U_{i}=\sum_{\mu =1}^{N_{\mathrm{neu}}}w_{\mu }^{\left(1\right)}\;\tanh\left(\sum_{\nu =1}^{N_{\mathrm{des}}}w_{\mu \nu }^{\left(0\right)}D_{\nu }^{i}-b_{\mu }^{\left(0\right)}\right)-b^{\left(1\right)}$$

where tanh­(*x*) is the activation function, *w* ^(0)^ represents the weight parameters connecting the input layer (with dimension *N*_des_) and the hidden layer (with dimension *N*_neu_), *w* ^(1)^ represents the weight parameters connecting the hidden layer and the output layer (the site energy), *b* ^(0)^ represents the bias parameters in the hidden layer, and *b* ^(1)^ represents the bias parameter in the output layer. All of these parameters are trainable.

**Figure 1:**
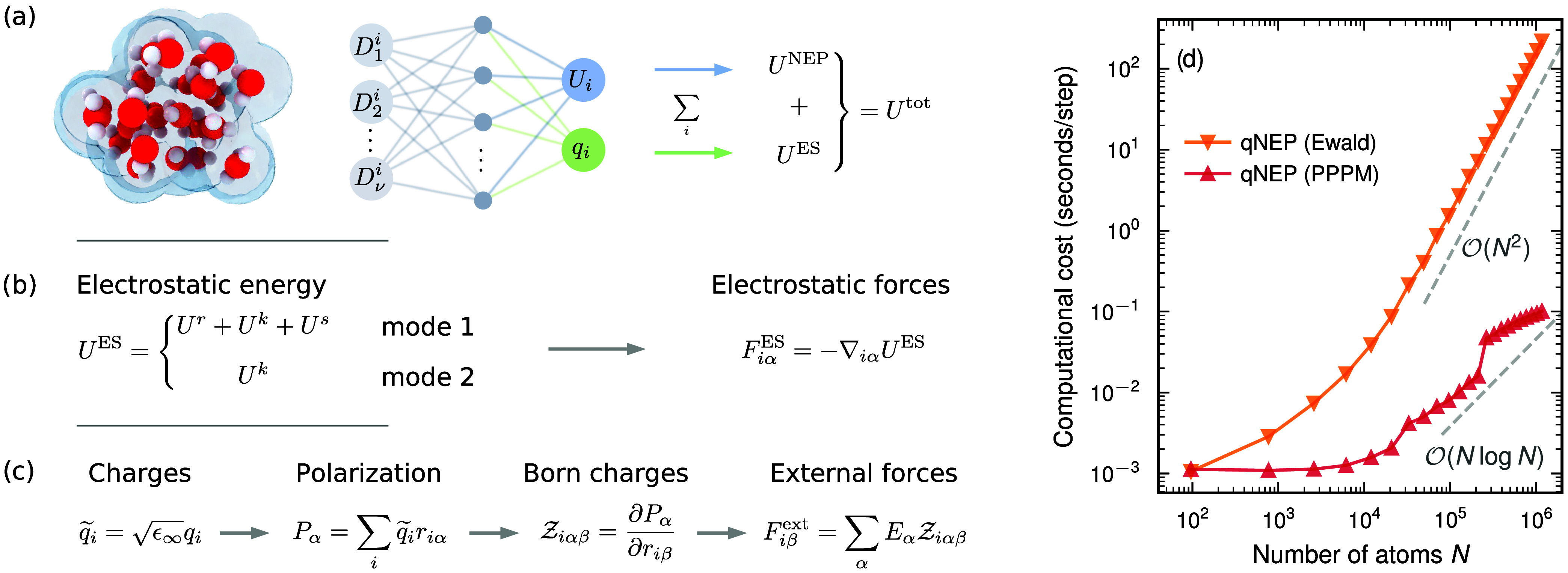
Schematic illustration of the qNEP framework. (a) Neural network architecture of a qNEP model with two outputs: a site energy *U*_*i*_ and a partial charge *q*_*i*_. The site energies are summed to yield the NEP contribution *U* ^NEP^ (Section 2.1) to the total energy *U* ^tot^, while interactions between the partial charges give rise to the electrostatic energy *U* ^ES^ (Section 2.2). (b) Evaluation of the electrostatic energy and corresponding forces **F**_*i*_^ES^ (Section 2.3), including either both real- and reciprocal-space contributions (mode 1) or the reciprocal-space contribution only (mode 2). (c) Derived response properties obtained from the partial charges, such as the polarization **P**, the Born effective charges $Z_{i}$ , and forces induced by external electric fields (Section 2.4). (d) Computational cost for evaluating the electrostatic contribution, comparing direct Ewald summation with the PPPM method (Section 2.6), which offers superior computational performance, particularly for large systems (shown here for water; Section 3.1).

The total energy is given by the sum of the site energies

2 $$U^{\mathrm{NEP}}=\sum_{i}U_{i}$$

The input layer corresponds to the descriptor vector **D** ^*i*^ (of dimension *N*_des_) for a given atom *i*, with its components denoted as *D*_ν_^*i*^ in eq 1. Similar to the symmetry functions used in the Behler–Parrinello approach,^1,31^ the descriptor components in NEP are classified into radial and angular ones. Both types of descriptors involve additional trainable parameters used to discriminate between atomic species. Details on the descriptor components and the associated trainable parameters can be found in refs 24,28 .

### 2.2 The qNEP Model Architecture

The qNEP approach extends the NEP framework by adding an additional output node to predict partial charges *q*_*i*_. Using separate NNs for the potential energy and the charges does not lead to a noticeable improvement in training accuracy, while increasing both the data requirements and the training cost. We therefore adopt a single-NN architecture (Figure 1a; blue and green output layers).

Given the partial charges, the electrostatic energy *U* ^ES^ is evaluated under periodic boundary conditions using the Ewald decomposition as the sum of three contributions,

3 $$U^{\mathrm{ES}}=U^{r}+U^{k}+U^{s}$$

where *U* ^r^ is the real-space component, *U* ^*k*^ is the reciprocal-space component (evaluated in *k*-space, hence the superscript *k*), and *U* ^s^ is the self-energy. The total energy in qNEP is given by the sum of the NEP energy and the electrostatic energy,

4 $$U^{\mathrm{tot}}=U^{\mathrm{NEP}}+U^{\mathrm{ES}}$$

We consider two different modes for evaluating the energy contribution associated with the partial charges. In mode 1, both the real-space and reciprocal-space contributions are included (Figure 1b), as adopted by Song et al.,^16^ and the electrostatic energy of the system is calculated according to eq 3. One may, however, argue that a short-ranged MLIP such as NEP already captures all short-range interactions, including the real-space component of electrostatics, making an explicit real-space electrostatic term potentially redundant. In mode 2, we therefore consider only the reciprocal-space contribution (Figure 1b), as adopted by Cheng et al.,^17,18^ i.e.,

5 $$U^{\mathrm{ES}}=U^{k}$$

One of the aims of this work is to evaluate the relative advantages and disadvantages of these two approaches in realistic systems.

In the remainder of this section, we present explicit expressions for the real-space and reciprocal-space contributions to the electrostatic energy, as well as for the self-energy. We then derive expressions for the force and virial (Section 2.3), as well as for the BEC tensor and related properties (Section 2.4). The latter enables coupling to external electric fields and the computation of quantities such as the dielectric function, infrared spectra, and ionic electrical conductivity.

During training of qNEP models, the NEP loss function is augmented with additional terms that penalize violations of charge conservation and, optionally, constrain the prediction of the BECs (Section 2.5). For the evaluation of reciprocal-space contributions, we implement both Ewald summation and the PPPM technique (Section 2.6). We have made functionality for training and deploying qNEP models available in the free-and-open-source GPUMD package^24^ from version 4.6, together with the supporting CALORINE Python package from version 3.3.^32^

#### 2.2.1 Real-Space Electrostatic Energy

The real-space electrostatic energy included in *U* ^ES^ when using mode 1 (eq 3) is given by

6 $$U^{r}=\frac{1}{2}\frac{1}{4\pi \epsilon_{0}}\sum_{i}\sum_{j\neq i}\frac{q_{i}q_{j}}{r_{\mathrm{ij}}}\mathrm{erfc}\left(\alpha ,r_{\mathrm{ij}}\right)$$

where erfc denotes the complementary error function, *r*_*ij*_ is the distance between atoms *i* and *j*, *q*_*i*_ is the charge of atom *i*, and ϵ_0_ is the vacuum permittivity. We use the terms “ion” and “atom” interchangeably, as it is conventional to use atom in the context of MLIPs, while partial charges are typically associated with ions. The real-space contribution is evaluated up to a cutoff radius *r*_c_.

The parameter α, which has the dimension of inverse length, controls the relative convergence rates of the real-space and reciprocal-space components. Larger values of α lead to faster convergence in real space with respect to the cutoff radius *r*_c_, whereas smaller values of α improve convergence in reciprocal space with respect to the cutoff wave vector *k*_max_ (see below). In this work, the real-space cutoff radius *r*_c_ is chosen to coincide with the pairwise cutoff of the associated NEP model, which typically lies in the range 4–8 Å. After fixing the NEP cutoff radius, we select α to provide sufficient accuracy for the real-space contribution. Specifically, we use α = π/*r*_c_, which is a conventional choice to converge the real-space contribution.^33^

#### 2.2.2 Reciprocal-Space Electrostatic Energy

The reciprocal-space contribution to the electrostatic energy, *U* ^*k*^, which is required in both mode 1 (eq 3) and mode 2 (eq 5), is given by

7 $$U^{k}=\frac{1}{4\pi \epsilon_{0}}\sum_{k\neq 0}^{k<k_{\max}}G\left(k\right)S\left(k\right)S^{*}\left(k\right)$$

where

8 $$S\left(k\right)\equiv \sum_{i}q_{i}\;e^{-ik\cdot r_{i}}=S^{*}\left(-k\right)$$

is the structure factor. Here, **r**_*i*_ denotes the position of atom *i*, **k** is a reciprocal-space wave vector given by integer combinations of the reciprocal lattice basis vectors, and *k* = |**k**|.

The function

9 $$G\left(k\right)\equiv \frac{2\pi }{\Omega }\frac{1}{k^{2}}e^{-k^{2}/4\alpha^{2}}$$

where Ω is the volume of the simulation cell, corresponds to the product of the Green’s function of the Coulomb potential and a Gaussian smoothing function. The summation over wave vectors **k** in eq 7 excludes the **k** = 0 term, which corresponds to the total charge of the system, and is truncated at a maximum magnitude *k*_max_. An accuracy of approximately 10^–5^, consistent with that of the real-space contribution, is achieved by choosing *k*_max_ = 2πα.

#### 2.2.3 Self-Energy

For mode 1 (eq 3), we also include the self-energy term, which removes the unphysical interaction of each charge with its own screening cloud

10 $$U^{s}=-\frac{1}{4\pi \epsilon_{0}}\frac{\alpha }{\sqrt{\pi }}\sum_{i}q_{i}^{2}$$

which is consistent with the approach adopted by Song et al.^16^

### 2.3 Energy Derivatives

Starting from the energy, one can derive other microscopic quantities, such as the force and virial. A crucial aspect in the present context is that both static and dynamic contributions of the charges must be taken into account when evaluating energy derivatives. Here, static charge refers to contributions originating from the explicit 1/*r* dependence in the Coulomb energy, whereas dynamic charge refers to contributions arising from the position dependence of the charges themselves.

qNEP models are many-body potentials, and general expressions for the force and virial of such potentials have been discussed previously.^34^ An important result is that Newton’s third law (in its weak form) continues to hold for many-body potentials. Accordingly, the force acting on atom *i* can be expressed as a pairwise summation^34^

11 $$F_{i}=\sum_{j\neq i}\left(F_{\mathrm{ij}}-F_{\mathrm{ji}}\right)$$

where **F**_*ij*_ can be interpreted as a “partial force” contribution.

Using the partial forces, the per-atom virial tensor can be written as^34^

12 $$W_{i}=\sum_{j\neq i}r_{\mathrm{ij}}⊗F_{\mathrm{ji}}$$

Throughout this work, we define

13 $$r_{\mathrm{ij}}\equiv r_{j}-r_{i}$$

as the distance vector pointing from atom *i* to atom *j*.

Analogous to the electrostatic energy, the force is evaluated using an Ewald decomposition into real-space, reciprocal-space, and self-energy contributions. For static charges, the real-space term gives rise to purely pairwise partial forces, while the reciprocal-space contribution is most naturally expressed as a per-atom force. When the charges depend on the atomic configuration, additional force contributions arise in all three parts of the Ewald sum through the chain rule, i.e., from terms proportional to (∂*E*/∂*q*_*i*_)­(∂*q*_*i*_/∂**r**). These dynamic-charge contributions can be cast into a partial-force form and combined consistently with the static terms, allowing the total force and virial to be evaluated within the same many-body framework.

We now present explicit expressions for the partial forces associated with the different energy contributions in the qNEP model. For the NEP contribution, the partial force can be written as

14 $$F_{\mathrm{ij}}^{\mathrm{NEP}}=\sum_{\nu =1}^{N_{\mathrm{des}}}\frac{\partial U_{i}}{\partial D_{\nu }^{i}}\frac{\partial D_{\nu }^{i}}{\partial r_{\mathrm{ij}}}$$

Details on the derivatives of the descriptors with respect to atomic positions can be found in previous work.^28^ For the electrostatic contribution, we discuss the three components separately in the following subsections.

For the contributions due to the dynamic charges, the per-atom virial is obtained from the corresponding partial forces according to eq 12.

#### 2.3.1 The Real-Space Contribution

For static charges, the real-space contribution to the electrostatic energy is purely two-body (pairwise) in nature. Nevertheless, it can be formulated within the general many-body potential framework introduced above. Within this framework, the partial force can be derived as

15 $$F_{\mathrm{ij}}^{r,\mathrm{static}}=\frac{1}{2}\frac{-q_{i}q_{j}}{4\pi \epsilon_{0}}\frac{r_{\mathrm{ij}}}{r_{\mathrm{ij}}^{3}}\left[\frac{2\alpha }{\sqrt{\pi }}r_{\mathrm{ij}}\;e^{-\alpha^{2}r_{\mathrm{ij}}^{2}}+\mathrm{erfc}\left(\alpha r_{\mathrm{ij}}\right)\right]$$

When the charges are dynamic, i.e., explicitly dependent on the atomic configuration, an additional contribution to the partial force arises from the position dependence of the charges. This contribution is given by

16 $$F_{\mathrm{ij}}^{r,\mathrm{dynamic}}=\frac{1}{4\pi \epsilon_{0}}\frac{\partial q_{i}}{\partial r_{\mathrm{ij}}}\left(\sum_{k\neq i}\frac{q_{k}}{r_{\mathrm{ik}}}\mathrm{erfc}\left(\alpha r_{\mathrm{ik}}\right)\right)$$

The derivative of the charge with respect to the relative position vector is evaluated using the chain rule

17 $$\frac{\partial q_{i}}{\partial r_{\mathrm{ij}}}=\sum_{\nu =1}^{N_{\mathrm{des}}}\frac{\partial q_{i}}{\partial D_{\nu }^{i}}\frac{\partial D_{\nu }^{i}}{\partial r_{\mathrm{ij}}}$$

#### 2.3.2 The Reciprocal-Space Contribution

The force acting on atom *i* due to the reciprocal-space contribution of the electrostatic energy with static charges can be derived to be

Fik,static=2qi4πϵ0∑k≠0k<kmaxkG(k)Im[S(k)eik·ri] 18

The partial force due to dynamic charges can be derived to be

Fijk,dynamic=2∑k≠0k<kmaxG(k)4πϵ0Re[S(k)∂qi∂rijeik·ri] 19

While the reciprocal-space contribution to the force due to static charges is usually not calculated in a pairwise manner, the virial can be calculated in a per-atom style,^35^

$$W_{i}^{k,\mathrm{static}}=\overset{k<k_{\max}}{\underset{k\neq 0}{\sum }}\frac{G\left(k\right)q_{i}\;e^{ik\cdot r_{i}}S\left(k\right)}{4\pi \epsilon_{0}}B$$ 20

Here, **B** is a *k*-space stress kernel that results from the derivative of the reciprocal-space electrostatic energy with respect to a homogeneous strain of the simulation cell and maps each **k**-mode contribution onto a second-rank virial tensor,

21 $$B=I-\left(\frac{2}{4\alpha^{2}}+\frac{2}{k^{2}}\right)K$$

where **I** is the 3 × 3 identity tensor and **K** is a tensor with components *K*_αβ_ = *k*_α_ *k*_β_. If the per-atom virial is not needed, the total virial can be more cheaply calculated as

22 $$W^{k,\mathrm{static}}=\sum_{k\neq 0}^{k<k_{\max}}\frac{G\left(k\right)|S\left(k\right){|}^{2}}{4\pi \epsilon_{0}}B$$

Note that although **B** is a 3 × 3 tensor, it has only six independent components, reflecting the symmetry of the stress tensor and the fact that the reciprocal-space contribution ultimately derives from an underlying pairwise electrostatic interaction.

#### 2.3.3 The Self-Energy Contribution

For static charges, the self-energy does not contribute to the force since it depends only on the fixed charge values and is therefore independent of the atomic positions. When the charges are configuration-dependent, however, the self-energy acquires an implicit position dependence through *q*_*i*_({**r**}), which gives rise to an additional force contribution. In this case, the corresponding partial force is

23 $$F_{\mathrm{ij}}^{s,\mathrm{dynamic}}=-\frac{2}{4\pi \epsilon_{0}}\frac{\alpha }{\sqrt{\pi }}q_{i}\frac{\partial q_{i}}{\partial r_{\mathrm{ij}}}$$

As in the other contributions, the derivative of the charge with respect to position is evaluated using the chain rule introduced above.

### 2.4 Born Effective Charge and Related Properties

The output charges *q* can be used to compute the macroscopic polarization **P** and the associated BECs (Figure 1c), as first discussed by Zhong et al.^19^ Before calculating the polarization, the learned partial charges need to be scaled^36^

24 $${\mathrm{q̃}}_{i}=\sqrt{\epsilon_{\infty }}q_{i}$$

where ϵ_∞_ denotes the high-frequency relative permittivity, also known as the electronic dielectric constant. Using these notations, the Coulomb potential between two charges can be written as

25 $$\frac{1}{4\pi \epsilon_{0}}\frac{q_{i}q_{j}}{r_{\mathrm{ij}}}=\frac{1}{4\pi \epsilon_{0}\epsilon_{\infty }}\frac{{\mathrm{q̃}}_{i}{\mathrm{q̃}}_{j}}{r_{\mathrm{ij}}}$$

This rescaling accounts for electronic screening effects that are not explicitly included in the ionic degrees of freedom. The learned partial charges *q*_*i*_ are thus screened charges, while the scaled charges *q̃*_*i*_ can be understood as naked charges. The high-frequency relative permittivity ϵ_∞_ is material-specific and is generally taken as a trainable parameter.

In this work, we treat ϵ_∞_ as a trainable parameter and adopt an isotropic form. Fixing ϵ_∞_ to a density-functional theory (DFT)-computed reference value is straightforward for homogeneous systems but becomes ill-defined in heterogeneous or multiphase systems, where different regions of the simulation cell are associated with different dielectric environments; treating it as a training parameter avoids this ambiguity. For homogeneous training sets, the fitted value of $\sqrt{\epsilon_{\infty }}$ is generally in reasonable agreement with the DFT result, as illustrated by the water models discussed in Section 3.1.

For nonperiodic systems, where absolute positions are well-defined, the polarization (which reduces to the dipole moment) can be written as

26 $$P_{\alpha }=\sum_{i}{\mathrm{q̃}}_{i}r_{i\alpha }$$

and the BEC tensors can be obtained as

Ziαβ=∂Pα∂riβ=q̃iδαβ+∑jNrjα∂q̃j∂riβ= q̃iδαβ−∑j≠i(riα∂q̃i∂rijβ−rjα∂q̃j∂rjiβ) 27

where the second expression follows from rewriting the derivatives in terms of relative position vectors. For periodic systems, absolute positions are not well-defined, and the polarization must be expressed in a translationally invariant form,

28 $$Z_{i\alpha \beta }={\mathrm{q̃}}_{i}\delta_{\alpha \beta }+\frac{1}{2}\sum_{j\neq i}\left(r_{\mathrm{ij}\alpha }\frac{\partial {\mathrm{q̃}}_{i}}{\partial r_{\mathrm{ij}\beta }}-r_{\mathrm{ji}\alpha }\frac{\partial {\mathrm{q̃}}_{j}}{\partial r_{\mathrm{ji}\beta }}\right)$$

Using the BEC, the force acting on ion *i* in response to an external electric field **E** can be written as

29 $$F_{i\beta }^{\mathrm{ext}}=\sum_{\alpha }E_{\alpha }Z_{i\alpha \beta }$$

During MD simulations, the time derivative of the polarization corresponds to the ionic electric current and can be evaluated from the BEC and the atomic velocities **v** as

30 $${Ṗ}_{\alpha }=\frac{dP_{\alpha }}{dt}=\sum_{i=1}^{N}\sum_{\beta }Z_{i\alpha \beta }v_{i\beta }$$

The polarization along the trajectory can then be obtained by time integration of *Ṗ*_α_, provided that the initial value is known. The Fourier transform of the time autocorrelation function (ACF) ⟨**Ṗ**(0)·**Ṗ**(*t*)⟩ is proportional to the infrared spectrum, while its time integral yields the ionic electrical conductivity

31 $$\sigma =\frac{1}{3k_{B}\mathrm{TV}}\int_{0}^{\infty }\left\langle Ṗ\left(0\right)\cdot Ṗ\left(t\right)\right\rangle dt$$

### 2.5 Training of the Models

All parameters in the descriptor and the NN for the potential energy and partial charges are trainable, including the high-frequency relative permittivity ϵ_∞_. As in the original NEP approach, these parameters are optimized using the SNES method.^30^ The optimization is guided by a loss function, which we denote as *L*(**z**), where the abstract vector **z** collects all trainable parameters.

The loss function is defined as a weighted sum of the root-mean-square error (RMSE) values for the energies (Δ_e_), forces (Δ_f_), virials (Δ_v_), BECs ( $\Delta_{Z}$ ), and total charges (Δ_Q_), together with $L_{1}$ and $L_{2}$ regularization terms,

32 $$L\left(z\right)=\lambda_{e}\Delta_{e}\left(z\right)+\lambda_{f}\Delta_{f}\left(z\right)+\lambda_{v}\Delta_{v}\left(z\right)+\lambda_{Z}\Delta_{Z}\left(z\right)+\lambda_{Q}\Delta_{Q}\left(z\right)+\lambda_{1}||z{\left|\right|}_{1}+\lambda_{2}||z{\left|\right|}_{2}^{2}$$

Here, Δ_Q_ refers to the total charge of each structure rather than to individual partial charges and is included to penalize violations of charge conservation. This penalty ensures that the predicted total charge deviates from the target value only marginally.

To enforce strict charge conservation or charge neutrality, a final total-charge correction is applied before evaluating the electrostatic energy and the BECs. Specifically, the scaled charges *q̃*_*i*_ in a system with *N* atoms are corrected as follows

33 $${\mathrm{q̃}}_{i}\to {\mathrm{q̃}}_{i}-\frac{1}{N}\sum_{i}\left(Q-{\mathrm{q̃}}_{i}\right)$$

where *Q* is the target total charge of the structure, which is zero in all the cases studied in this work. With the penalization term λ_Q_Δ_Q_(**z**) in the loss function, the total charge of the structure is already close to the target Q and the correction above mainly serves to ensure strict charge conservation that can be important in, e.g., simulations with an external electric field.

The inclusion of target BECs in the loss function is optional, and reference data need only be provided for a subset of the training structures. This makes it possible to limit the number of reference BEC calculations, which are computationally more demanding than calculations of energies, forces, or virials.

In practice, convergence of the BEC predictions can be achieved with a relatively small number of reference BEC calculations. For example, for BaTiO_3_, we find that approximately 64 structures (less than 4% of the full training set) are sufficient (Figure S11).

### 2.6 Accelerated Calculation of the Reciprocal-Space Contribution Using PPPM

In the preceding sections, we assumed a direct Ewald summation for evaluating the reciprocal-space contribution to the electrostatic energy. In practical simulations, however, the use of fast Fourier transform (FFT)-based methods can significantly reduce the computational cost.^37^ This leads to particle–mesh approaches such as PPPM,^25^ particle–mesh Ewald (PME),^38^ and smooth PME (SPME),^39^ which are closely related and can be mathematically transformed into one another.^40,41^ Here, we adopt the PPPM method and extend it to consistently account for both static and dynamic partial charges.

Within the PPPM framework, the reciprocal-space contribution to the electrostatic energy retains the formal structure of eq 7, but the Green’s function factor *G*(*k*) is replaced by an optimized counterpart, *G* ^opt^(**k**),^37^

34 $$U^{k}=\frac{1}{4\pi \epsilon_{0}}\sum_{k\neq 0}G^{\mathrm{opt}}\left(k\right)S\left(k\right)S^{*}\left(k\right)$$

The structure factor *S*(**k**) is evaluated on a regular mesh of dimension *N*_*x*_ × *N*_*y*_ × *N*_*z*_. Mesh charges are obtained by interpolating the original partial charges using a charge assignment function *W*(**r**_*i*_ – **r**_s_),^37^ which specifies the fraction of the charge at position **r**_*i*_ assigned to the mesh point **r**_s_. The charge assignment function can be decomposed into the three Cartesian directions,

35 $$W\left(r_{i}-r_{s}\right)=W\left(x_{i}-x_{s}\right)W\left(y_{i}-y_{s}\right)W\left(z_{i}-z_{s}\right)$$

Explicit expressions for the charge assignment functions for interpolation orders *P* = 1 to *P* = 7 are given by Deserno and Holm.^40^

In our implementation, we use a mesh spacing smaller than 1 Å together with an interpolation order *P* = 5, which yields an accuracy of approximately 10^–4^. The corresponding optimized Green’s function is given by^37,41^

36 $$G^{\mathrm{opt}}\left(k\right)=\frac{G\left(k\right){\left[\prod_{\alpha =1}^{3}{\mathrm{sinc}}^{P}\left(\frac{\pi n_{\alpha }}{N_{\alpha }}\right)\right]}^{2}}{\prod_{\alpha }^{3}\left(1-\frac{5}{3}z_{\alpha }^{2}+\frac{7}{9}z_{\alpha }^{4}+\frac{17}{189}z_{\alpha }^{6}+\frac{2}{2835}z_{\alpha }^{8}\right)}$$

where sinc­(*x*) = sin­(*x*)/*x*, *z*_α_ = sin­(π*n*_α_/*N*_α_), and *n*_α_ are integer mesh indices satisfying −*N*_α_/2 ≤ *n*_α_ < *N*_α_/2.

The reciprocal-space force due to static charges can be computed in several equivalent ways.^37^ Here, we employ the *i* **k**-differentiation scheme,

$$F_{i}^{k,\mathrm{static}}=-\frac{2}{4\pi \epsilon_{0}}q_{i}\underset{r_{s}}{\sum }W\left(r_{i}-r_{s}\right)F^{-1}\left[ikS\left(k\right)G^{\mathrm{opt}}\left(k\right)\right]$$ 37

For dynamic charges, the force contribution arises from the explicit position dependence of the charges and is evaluated using an analytical differentiation scheme,

38 $$F_{\mathrm{ij}}^{k,\mathrm{dynamic}}=\frac{2}{4\pi \epsilon_{0}}\frac{\partial q_{i}}{\partial r_{\mathrm{ij}}}\sum_{r_{s}}W\left(r_{i}-r_{s}\right)F^{-1}\left[S\left(k\right)G^{\mathrm{opt}}\left(k\right)\right]$$

For the reciprocal-space contribution to the virial arising from static charges, the total virial can be evaluated in a manner analogous to eq 22. If a per-atom decomposition is required, the per-atom virial can be written as

39 $$W_{i}^{k,\mathrm{static}}=\frac{q_{i}}{4\pi \epsilon_{0}}\sum_{r_{s}}W\left(r_{i}-r_{s}\right)F^{-1}\left[S\left(k\right)G^{\mathrm{opt}}\left(k\right)B\right]$$

A forward FFT is used to compute *S*(**k**) from the charge mesh, while backward FFTs are used to evaluate the forces and virials, as indicated by the $F^{-1}$ operations. Specifically, three backward FFTs are required to obtain the three Cartesian components of the force due to static charges according to eq 37, and one backward FFT is required to obtain the force contribution due to dynamic charges according to eq 38. If the per-atom virial is needed, six backward FFTs are required to evaluate the six independent components of the virial tensor according to eq 39. Efficient implementations of these operations can be realized using standard libraries from the CUDA and HIP toolkits.

The resulting PPPM implementation exhibits an overall O(Nlog⁡N) scaling with the number of atoms due to the use of FFTs, in contrast to the quadratic scaling of the direct Ewald summation. In practice, the PPPM method features a small prefactor and near-linear scaling over the system sizes considered here, resulting in a computational cost that is one to several orders of magnitude lower than for the Ewald approach (Figure 1d).

## 3 Results

To illustrate the potential of the qNEP approach, we constructed models for several distinct classes of materials and employed them in prototypical applications. In the following section (Section 3.1), we consider water as a representative liquid system. We show that the inclusion of electrostatics in qNEP models systematically improves accuracy compared with regular NEP models at only a modest additional computational cost. The resulting models enable simulations of water systems comprising hundreds of thousands or even millions of atoms and allow for simulation times of several tens of nanoseconds per day on a single GPU. We further demonstrate the capability of qNEP models to predict the infrared spectrum of water as a function of temperature.

We then turn to two crystalline systems, again observing systematic improvements upon including electrostatics. First, for the prototypical ionic conductor Li_7_La_3_Zr_2_O_12_, we show that the temperature dependence of the structural parameters and the transition from the low-temperature tetragonal phase to the high-temperature cubic phase are in close agreement with experimental data (Section 3.2). Further analysis reveals a qualitative change in the charge distribution across the phase transition, which is reflected in the ionic electrical conductivity, with the activation energy decreasing from 1.45 eV in the tetragonal phase to 0.29 eV in the cubic phase.

Next, we consider the prototypical ferroelectric BaTiO_3_, demonstrating that qNEP models readily reproduce not only the experimentally observed phase transitions and structural changes, but also the associated evolution of the polarization (Section 3.3). We map out polarization–electric field (poling) loops at different temperatures, illustrating the coupling to external electric fields. In addition, we extract the temperature dependence of both the dielectric function and the dielectric constant.

Finally, we examine magnesium corrosion in aqueous media, a reactive solid–liquid interface that combines metallic and insulating components (Section 3.4). The qNEP approach captures the diverse, environment-dependent charge states present in this system and, owing to its computational efficiency, enables simulations of the conversion of metallic Mg into hydroxylated and solvated species under highly reactive conditions over time scales of many nanoseconds.

### 3.1 Liquid Water

Water is a representative liquid system in which the electrostatics between the components plays an important role. To train models, we employed the data set of Zhang et al.^44^ as curated by Xu et al.,^42^ who provided a split into 1388 training and 500 validation structures. All structures contain 384 atoms, and energies, forces, and stresses were obtained from DFT calculations using the strongly constrained and appropriately normed semilocal density-functional (SCAN) exchange–correlation functional^45^ (see refs 42,44 for details). In addition, to enable learning of the dielectric response, we computed BECs for 194 structures randomly selected from the original data set (see Supporting Note 4 for details).^46–48^ We trained one NEP model and two qNEP models (one for each electrostatic mode) using identical hyperparameters (Supporting Note 3, Figure S1).

The RMSEs demonstrate a systematic improvement in the accuracy of energies, forces, and stresses for the qNEP models compared to those for the NEP model (Figure 2a), highlighting the importance of long-range electrostatic interactions in water. The two qNEP variants perform very similarly with the model trained using only the reciprocal-space contribution (mode 2) yielding marginally lower errors for energies and forces. This trend, which is also observed for the other systems discussed below, suggests that explicitly including short-ranged electrostatic interactions may be redundant when such interactions are already captured by the underlying short-ranged MLIP. Both qNEP models accurately reproduce the BECs (Figure 2b,c; see also Figure S2 for the mode 1 model).

**Figure 2:**
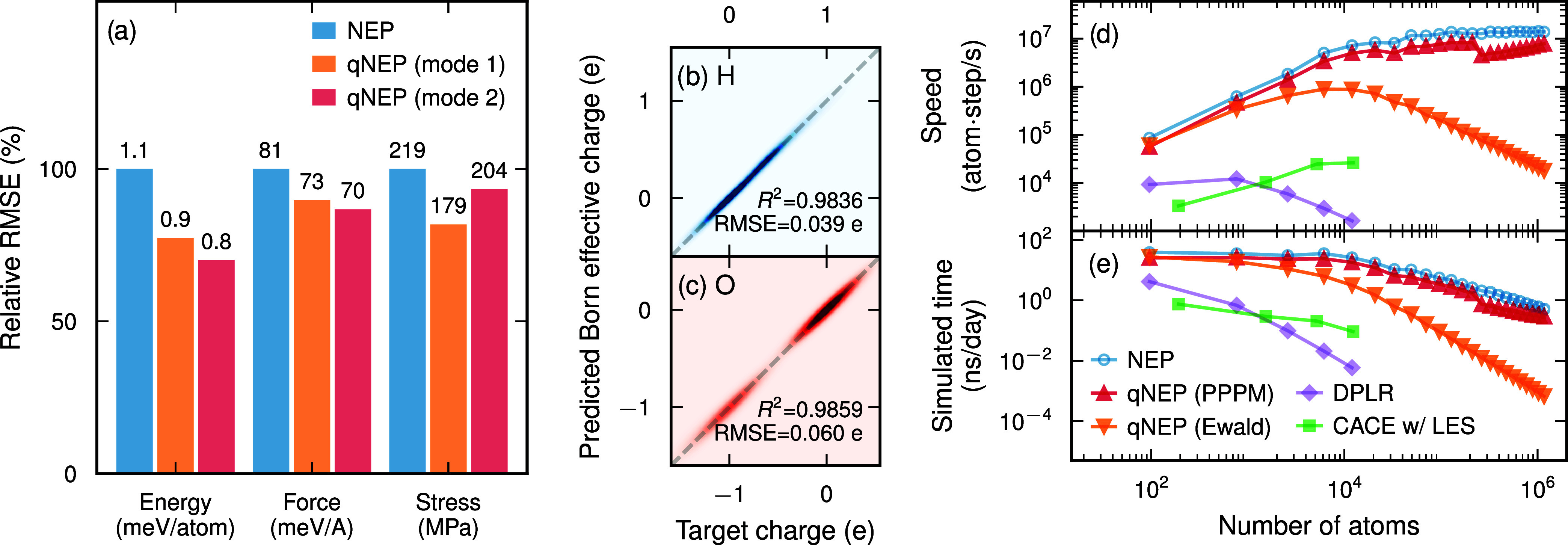
Performance of qNEP for water. (a) Relative validation RMSEs of NEP and qNEP models. (b, c) Parity plots of Born effective charges for H and O, respectively, obtained from the qNEP model trained on the full reference data set using mode 2. (d, e) Performance comparison of qNEP models with NEP, a CACE model with LES, and a deep potential model with long-range electrostatic interactions (DPLR), in terms of (d) computational speed and (e) simulated time achievable within one day on a single Nvidia RTX 4090 GPU using a time step of 0.5 fs.

The qNEP models also learn the square root of the high-frequency dielectric constant, $\sqrt{\epsilon_{\infty }}$ , which appears in eq 33 and corresponds to the refractive index at optical frequencies, *n*. Although $\sqrt{\epsilon_{\infty }}$ primarily acts as a hyperparameter during training, it is noteworthy that the fitted values, $\sqrt{\epsilon_{\infty }}=n=1.77$ and 1.53 for modes 1 and 2, respectively, are in reasonable agreement with the experimental value of 1.33 at ambient conditions.^49,50^ Such a comparison is meaningful here because ϵ_∞_ can be expected to be relatively homogeneous over the training domain.

Computational efficiency is critical for production simulations. The NEP model achieves speeds exceeding 10^7^ atom step/s on a single consumer-grade GPU (Nvidia RTX 4090) for systems containing at least 10^4^ atoms (Figure S4). Using a time step of 0.5 fs, this corresponds to up to 40 ns of MD simulation per day (Figure 2e; see also Figure S4 for results on other GPUs). When employing qNEP models together with the PPPM method, the computational cost increases only by about a factor of 2 (Figures 2e and S5). These numbers are several orders of magnitude higher than those achievable with, for example, a CACE model with LES^17^ or a deep potential model with long-range electrostatic interactions.^8^

As an additional validation, we computed partial radial distribution functions from both classical and path integral molecular dynamics (PIMD) simulations (Figure 3a,b; Supporting Note 5).^51,52^ The results are essentially indistinguishable between the NEP and qNEP models and are in very good agreement with ab initio MD (AIMD) simulations performed using the same exchange–correlation functional.^42^

**Figure 3:**
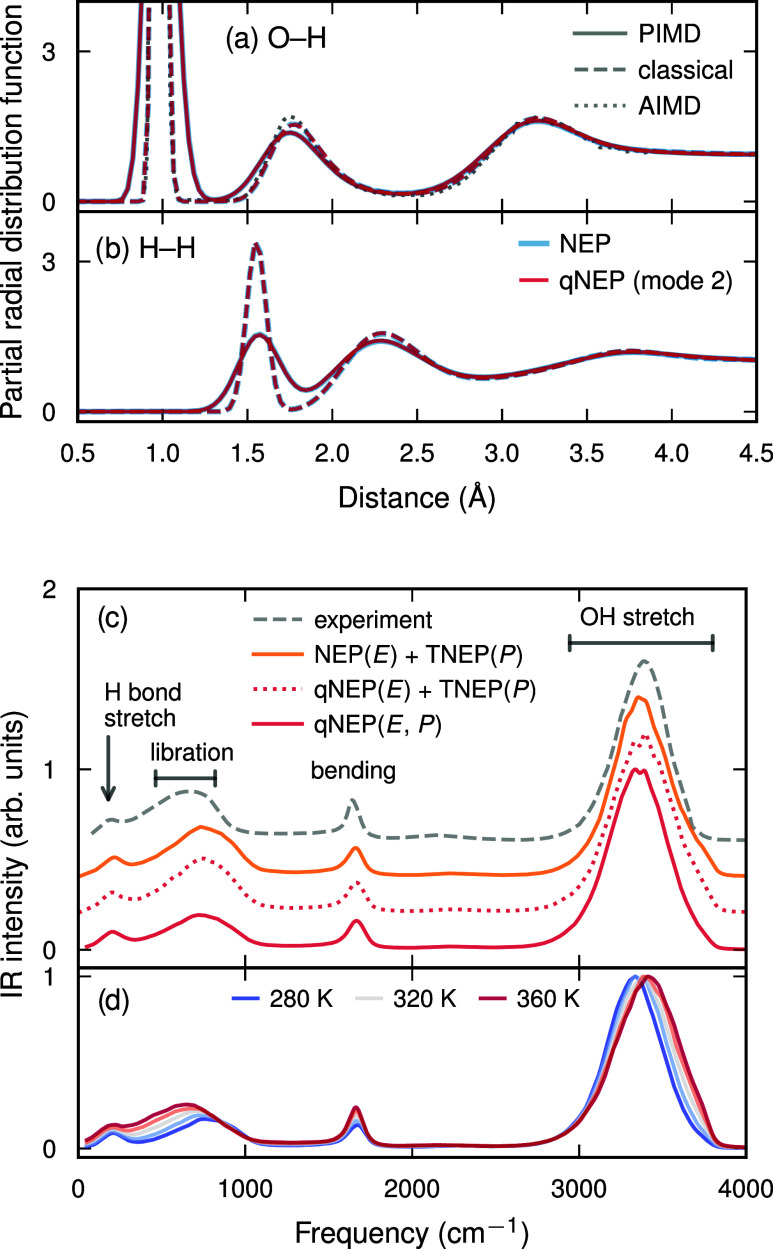
Properties of water with NEP and qNEP models. (a, b) Partial radial distribution functions for (a) O–H and (b) H–H pairs in liquid water at 300 K. Solid and dashed lines correspond to quantum simulations performed using PIMD and classical simulations using standard MD, respectively, for the NEP (blue) and qNEP (red) models. Classical ab initio MD (AIMD) reference data (dotted line) from ref 42 are included for comparison. (c, d) Infrared spectra obtained from classical MD simulations via the time ACF of the ionic electric current **Ṗ**. (c) Spectra obtained using different combinations of the NEP and qNEP (mode 2) models for sampling the energy landscape (*E*) and the TNEP (from ref 43) and qNEP models for computing the polarization (*P*) in comparison with experiment. (d) Temperature dependence of infrared spectra obtained using the qNEP model (mode 2) for both *E* and *P*.

Finally, the availability of BECs combined with the high computational efficiency of qNEP models enables straightforward calculation of infrared spectra from the time ACF of the polarization or its time derivative^43^ (see Supporting Note 5 for details). At room temperature, the resulting infrared spectra compare well with experimental reference data,^53,54^ with remaining deviations attributable to the underlying exchange–correlation functional (Figure 3c). Upon increasing the temperature, we observe a blueshift of the O–H stretching band and a redshift of the librational band (Figure 3d), both of which can be attributed to a weakening of intermolecular vibrational coupling.

### 3.2 Lithium Lanthanum Zirconate Crystal

Garnet-type LLZO is among the most promising solid electrolyte materials for next-generation all-solid-state lithium batteries, combining high ionic conductivity with excellent chemical and electrochemical stability against lithium metal.^56^ Its garnet structure consists of a mobile Li-ion sublattice embedded within a rigid three-dimensional framework formed by interconnected LaO_8_ dodecahedra and ZrO_6_ octahedra, which create fast diffusion pathways for Li ions. As an ionic crystalline solid electrolyte, LLZO represents an ideal test case for assessing the importance of incorporating charge information into MLIPs, since electrostatic interactions between charged species play a central role in ionic transport. In addition, LLZO undergoes a well-known temperature-driven phase transition at approximately 900 K, from a low-temperature tetragonal phase (t-LLZO, *I*4_1_/*acd*, ITC number 142) to a high-temperature cubic phase (c-LLZO, *Ia*3̅*d*, ITC number 230). This transition is accompanied by an increase in the lithium ionic conductivity by several orders of magnitude^57^ and has an order–disorder character, involving a reorganization of the Li-ion sublattice from an ordered arrangement with fully occupied sites in t-LLZO to a disordered state with partially occupied sites in c-LLZO. The combination of strong electrostatic interactions and complex structural phase behavior makes LLZO a demanding and representative benchmark for evaluating the qNEP approach.

We trained NEP and qNEP models (Supporting Note 6) using the data set of Yan and Zhu,^57^ which comprises 1978 configurations of pristine LLZO with energies, forces, and stresses computed using the PBEsol exchange–correlation functional.^58^ Consistent with the trends observed for liquid water, the qNEP models reduce the RMSEs for energies, forces, and stresses by approximately 20–30% relative to the regular NEP model (Figure 4a; see also Figure S6).

**Figure 4:**
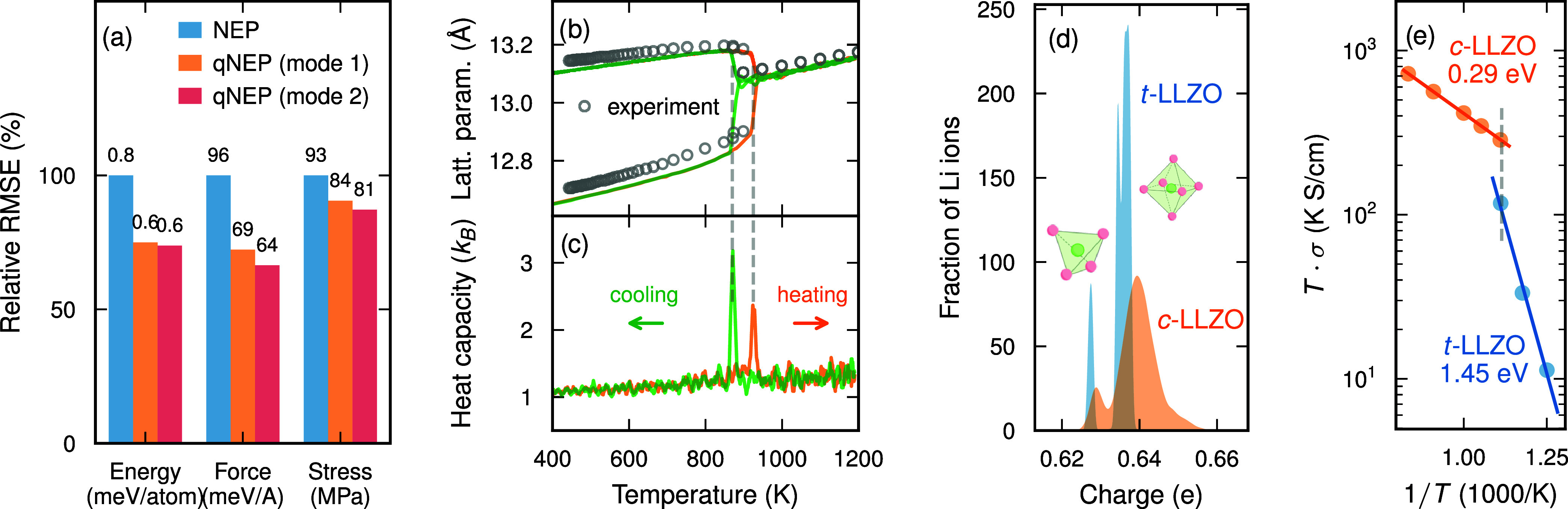
Garnet-type lithium lanthanum zirconate Li_7_La_3_Zr_2_O_12_ (LLZO). (a) Relative training RMSEs of NEP and qNEP models, with RMSE values reported above the corresponding columns. (b, c) Temperature dependence of (b) lattice parameters and (c) heat capacity obtained from heating and cooling simulations. Vertical dashed lines indicate the corresponding phase transition temperatures. Experimental data for the lattice parameters from ref 55. (d) Distribution of Li partial charges in the tetragonal (t-LLZO) and cubic (c-LLZO) phases after structural relaxation. The left-hand peaks in the distributions correspond to Li ions occupying tetrahedral sites (see inset), while the right-hand peaks correspond to octahedral sites (see inset). (e) Arrhenius plot of the ionic conductivity σ multiplied by temperature *T*. Activation energies extracted for the tetragonal and cubic phases are indicated. The vertical dashed line marks the average transition temperature of 900 K obtained from the heating and cooling runs.

Using the qNEP model trained in mode 2, we investigated the temperature dependence of the LLZO structure through heating and cooling simulations performed at a rate of 50 K/ns (Supporting Note 7).^59^ The resulting lattice parameters, and in particular the thermal expansion, are in good agreement with experimental measurements over the full temperature range considered^55^ (Figure 4b). Our simulations capture the phase transition from t-LLZO to c-LLZO at approximately 900 K, which is in excellent agreement with experimental observations. Additionally, a hysteresis of about 55 K is observed between the heating and cooling cycles (Figure 4c).

The environment-dependent dynamic charges predicted by the qNEP model enable a detailed analysis of the order–disorder transition in LLZO. We relaxed snapshots extracted from MD trajectories of both t-LLZO and c-LLZO and evaluated the distributions of Li-ion charges in each phase (Figure 4d). In t-LLZO, the charge distribution exhibits a lower-charge peak associated with Li ions occupying tetrahedral sites (Wyckoff position 8a) and a higher-charge peak corresponding to ions in octahedral sites (16f and 32g). The octahedral contribution further displays a split structure, which we attribute to the distinct occupations of the 16f and 32g Wyckoff sites, both of which are octahedrally coordinated but feature slightly different local environments. In c-LLZO, the charge distribution becomes broader, reflecting the disordered nature of the Li-ion sublattice in the cubic phase.

These structural and charge-distribution differences between t-LLZO and c-LLZO are directly reflected in the transport properties (Figure 4e). In particular, the ionic conductivity exhibits a pronounced reduction in activation energy, decreasing from 1.45 eV in t-LLZO to 0.29 eV in c-LLZO. The latter value is in good agreement with experimental data in the high-temperature region.^64^ The activation energy in t-LLZO is known to be highly sensitive to composition,^57^ whence a direct comparison is not possible but a possible target for future work.

### 3.3 Barium Titanate

Next, we consider the prototypical ferroelectric perovskite BaTiO_3_, which provides an ideal test case for evaluating qNEP models in the presence of strong electromechanical coupling and external electric fields. At low temperatures, BaTiO_3_ adopts a rhombohedral structure with an instantaneous polarization along the ⟨111⟩ direction due to off-centering of the Ti atoms.^60,67^ Upon heating, it undergoes a sequence of phase transitions to orthorhombic and tetragonal phases at 183 and 278 K, respectively, with polarization along the ⟨011⟩ and ⟨001⟩ directions.^60^ At temperatures above 393 K, the material becomes paraelectric and cubic.

NEP and qNEP models were trained using a data set extended from Lindgren et al.^68^ to 1832 structures, 1193 of which also included BECs (Supporting Note 8). Energies, forces, and stresses were obtained from DFT calculations^46^ using the r2SCAN exchange–correlation functional,^69^ while BECs were obtained using the PBEsol functional^58^ (Supporting Note 9). As in previous cases, the qNEP models achieve a higher accuracy than the corresponding NEP model. The qNEP model based on mode 1 and used for the simulations below achieves RMSEs of 1.2 meV/atom, 65 meV/Å, and 112 MPa for energies, forces, and stresses, respectively (Figure S9). For comparison, the corresponding RMSEs for the NEP model are 1.0 meV/atom, 70 meV/Å, and 136 MPa. Both qNEP models also perform very well at predicting the BECs (Figure S10).

The trained model correctly reproduces the sequence of four phases and yields transition temperatures of 151, 235, and 390 K, in good agreement with experiment (Figure 5a; Supporting Note 10). This agreement can be attributed to the accuracy of the underlying exchange–correlation functional, whose energetics are faithfully reproduced by the qNEP model. A hysteresis of up to 50 K is observed between heating and cooling runs, even at a comparatively low rate of 10 K/ns used here. This behavior reflects the first-order nature of the phase transitions despite their relatively small latent heats.

**Figure 5:**
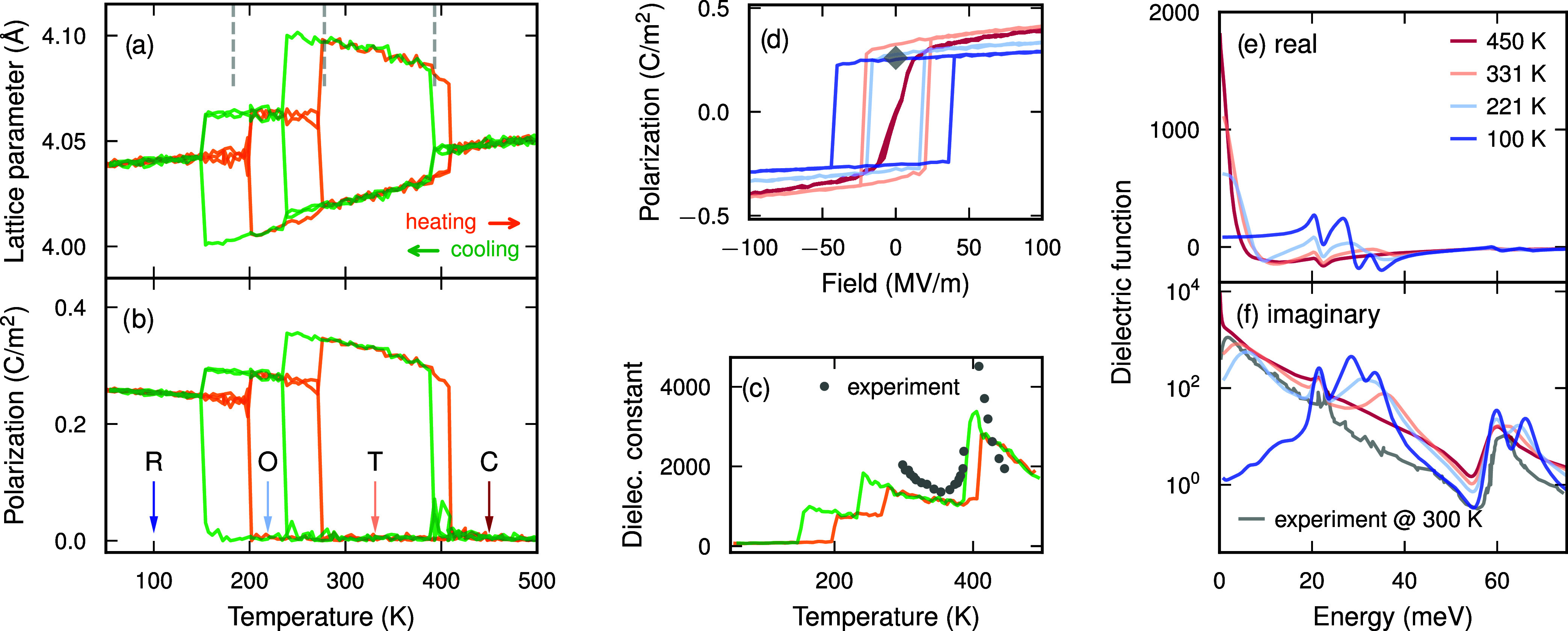
Ferroelectricity and dielectric response in barium titanate (BaTiO_3_). (a–c) Temperature dependence of (a) lattice constant, (b) polarization, and (c) dielectric constant during heating and cooling, revealing the sequence of phase transitions from the rhombohedral (R) ground state through the orthorhombic (O) and tetragonal (T) phases to the high-temperature cubic (C) phase. Vertical dashed lines indicate the experimentally observed transition temperatures.^60^ Experimental data points in panel (c) from ref 61. (d) Polarization–electric field (*P*–*E*) hysteresis loops and (e, f) real and imaginary parts of the dielectric function at different temperatures, corresponding to all four phases (indicated by arrows in panel (b)). The gray diamond in panel (d) marks the experimental value for the spontaneous polarization at room temperature^62^ and the gray line in panel (f) denotes an experimentally measured dielectric function at room temperature.^63^

Using the predicted BECs, we directly computed polarization as a function of temperature (Figure 5b). All four phases are clearly resolved, with a pronounced polarization at low temperatures and a vanishing polarization above 407 K during heating and 390 K during cooling. The polarization increases when progressing from the rhombohedral through the orthorhombic to the tetragonal phase, in agreement with both shell-model simulations^70^ and experimental measurements.^60^

For selected temperatures representative of each phase, we further applied external electric fields to map out polarization–electric field (*P*–*E*) hysteresis loops (Figure 5d; Supporting Note 10). As expected, the polarization can be switched by the applied field, and the spontaneous polarization in the absence of a field agrees well with the experimental value at room temperature^62^ (indicated by the gray diamond in Figure 5d). The coercive field depends sensitively on the switching frequency. Here, a frequency of 500 MHz was employed, which is low for MD simulations but still significantly higher than experimentally accessible frequencies.^71^ A direct quantitative comparison of coercive fields is, therefore, not meaningful. Nevertheless, the computational efficiency of the qNEP approach opens the door to detailed investigations of switching mechanisms and domain-wall motion, particularly within multiscale simulation frameworks.^72^

As a further validation, we computed the ionic contribution to the dielectric function from the time ACF of the ionic electric current (Supporting Note 10). The results reveal a strong dependence of the dielectric response on both the temperature and frequency (Figure 5e,f). Resonances appearing in the range 20–40 meV can be attributed to longitudinal optical modes at the Γ point. Their pronounced temperature dependence reflects the strong anharmonicity of BaTiO_3_, which also gives rise to the very large dielectric constant observed (Figure 5c; see also the static limit of the real part in Figure 5e). The maximum dielectric constant obtained here reaches values of approximately 3000 on the high-temperature side of the tetragonal–cubic-phase boundary, and is in good agreement with experimental measurements both in terms of magnitude and temperature dependence.^61^ In this context, it is important to emphasize that the present simulations only include the ionic (vibrational) contribution, while the experimental measurements also contain contributions from processes such as space-charge effects and grain boundaries. These occur on much longer time scales, and their relative importance depends on sample preparation.

Finally, we examine the effect of long-range electrostatics on the phonon dispersion in the cubic phase. The harmonic phonon dispersions predicted by the NEP and qNEP models, calculated with PHONOPY,^73,74^ agree closely over most of the Brillouin zone, except in the vicinity of the Γ point (Figure 6a; Supporting Note 11). This difference arises from long-range Coulomb interactions, which lead to a splitting between longitudinal (LO) and transverse optical (TO) phonon branches. Capturing this LO–TO splitting in the harmonic dispersion requires the application of nonanalytic corrections (NACs), which depend on knowledge of the BECs and are therefore only accessible within the qNEP framework.

**Figure 6:**
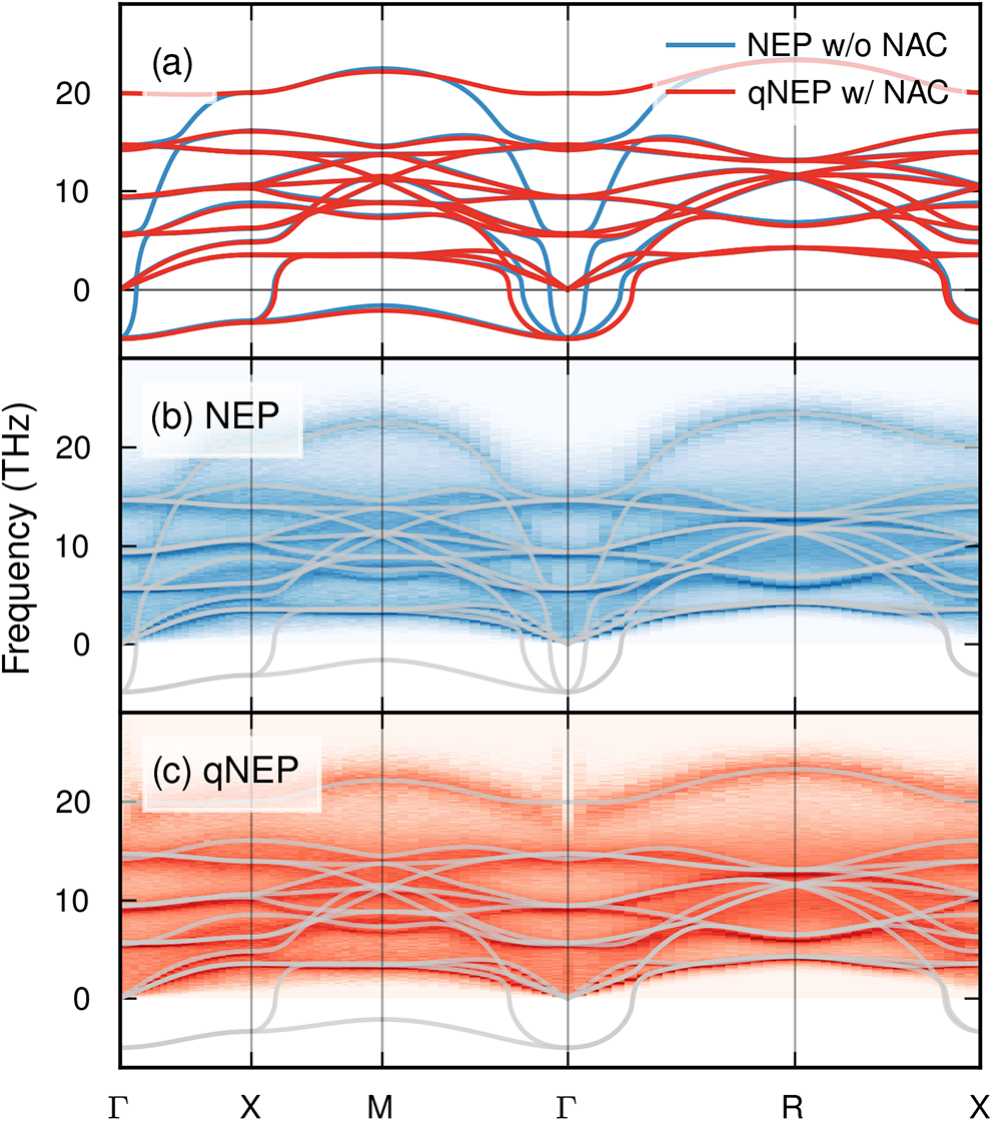
Phonons and LO–TO splitting in BaTiO_3_. (a) Harmonic phonon dispersion of the cubic phase obtained from NEP without nonanalytic correction (NAC) and from qNEP including NAC, illustrating the longitudinal–transverse optical (LO–TO) splitting. Imaginary phonon frequencies are shown as negative values and indicate dynamically unstable modes. (b, c) Spectral energy density obtained from (b) NEP and (c) qNEP at 500 K in the cubic-phase region calculated with DYNASOR.^65,66^ Gray lines reproduce the corresponding harmonic phonon dispersions shown in panel (a).

The same behavior is observed in finite-temperature phonon dispersions obtained from the spectral energy density^75^ using DYNASOR^65,66^ (Figure 6b,c): the LO and TO branches coincide at Γ for the NEP model but remain clearly separated for qNEP. Importantly, the LO–TO splitting in the qNEP spectral energy density emerges directly from the long-range electrostatic interactions treated explicitly during the MD simulations, without requiring any NACs, demonstrating a key advantage of the qNEP framework.

### 3.4 Corrosion of Magnesium in Water

Magnesium corrosion in aqueous media provides a prototypical example of a chemically reactive system in which the accurate treatment of charge transfer is essential. Changes in the Mg valence state directly govern the reaction pathways, intermediate species, and final corrosion products. In particular, the widely discussed incomplete-film monovalent magnesium-ion mechanism invokes the presence of a transient Mg^+^ intermediate and remains controversial.^76^ Resolving this issue is central to understanding the anomalous hydrogen evolution observed during magnesium corrosion and to guiding the design of corrosion-resistant magnesium alloys.

In previous studies, standard MLIPs were unable to explicitly represent evolving charge states and their associated long-range electrostatic interactions. This limitation hindered detailed investigations of charge evolution during the formation of corrosion intermediates and the dissolution dynamics of magnesium. The magnesium-water solid-liquid interface therefore constitutes a stringent and representative test case for qNEP models, as it directly probes their ability to capture environment-dependent charge transfer in reactive chemical processes.

To train NEP and qNEP models, we employed the reference data set of Liu et al.,^77^ which systematically covers magnesium dissolution mechanisms (see Supporting Note 12 for details). The qNEP model achieves RMSEs values of 12 meV/atom for energies, 198 meV/Å for forces, and 824 MPa for stresses, representing a clear improvement over the NEP model, which yields 14 meV/atom, 230 meV/Å, and 787 MPa, respectively (Figure S12). Crucially, the qNEP model successfully learns the relationship between local atomic environments and charge distributions (Figure 7a). The predicted charges clearly distinguish different chemical states: Mg atoms in the metallic bulk exhibit charges close to zero, while Mg atoms in hydroxides and oxides carry positive charges. Oxygen atoms associated with water molecules, surface hydroxyl groups, and oxides likewise show distinct charge signatures.

**Figure 7:**
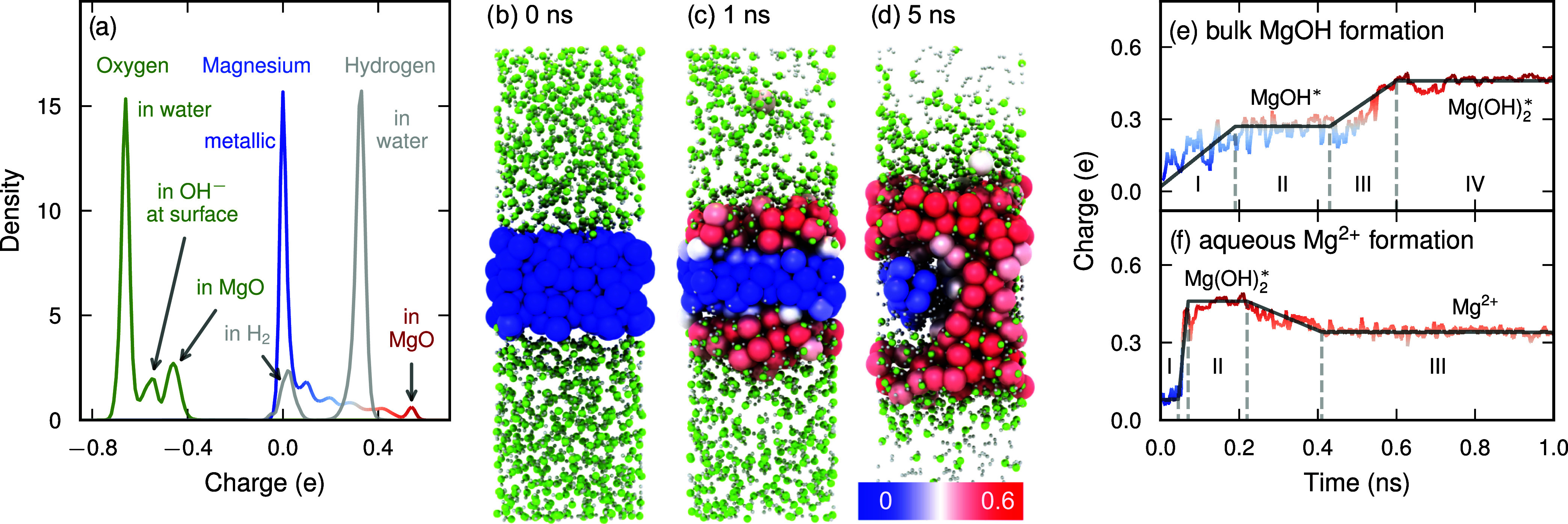
Corrosion of magnesium at a Mg–water interface. (a) Distribution of atomic charges in the reference data set, distinguishing Mg, O, and H species across different chemical environments. (b–d) Representative snapshots from an MD simulation illustrating the conversion of metallic Mg (large blue spheres) into oxidized Mg species (large red spheres). The color scale (shown in panel (d)) indicates the Mg charge state. Oxygen and hydrogen atoms are shown as small green and gray spheres, respectively. (e, f) Two characteristic charge-evolution pathways of Mg: (e) formation of bulk magnesium hydroxide and (f) dissolution into aqueous Mg^2+^ ions. Asterisks (*) denote adsorbed species.

MD simulations using the qNEP model therefore can not only capture the structural evolution of the Mg–water interface but also provide direct insight into transitions between different charge states. As a representative example, we consider the evolution of a highly reactive stepped Mg surface^78^ in contact with water at 700 K over a simulation time of 5 ns that was recently analyzed using a NEP model (Figure 7b–d). Detailed trajectories for such reactive systems are determined by a series of rare events, leading to a considerable variety between individual simulations. Here, rather than repeating the comprehensive analysis from ref 77, we therefore applied our qNEP model to analyze a specific trajectory from this earlier work (see Supporting Note 13 for details). We note that this trajectory was generated using the charge-unaware NEP model of ref 77; the qNEP model is applied here as a post hoc analysis tool to evaluate the evolution of the charge distribution. A systematic comparison of NEP and qNEP trajectories, which would quantify the effect of long-range electrostatics on the structural dynamics of this reactive interface, is a natural direction for future work.

After 1 ns (Figure 7c), the system clearly separates into an upper hydroxide layer (red), a lower metallic Mg substrate (blue), and a transitional interfacial region containing partially hydroxylated Mg species (light gray/pink). In addition, a small number of Mg atoms undergo oxidation via dissolution into the aqueous phase. As corrosion proceeds, most metallic Mg is converted into hydroxylated species within 5 ns (Figure 7d). While Mg atoms within the bulk metal and the corrosion product layer retain relatively stable valence states, atoms in the interfacial region exhibit distinct intermediate charge states corresponding to partial hydroxylation.

A detailed analysis of the charge trajectories reveals two characteristic corrosion pathways. The first pathway corresponds to the formation of solid-state corrosion products (Figure 7e). Here, Mg atoms at the interface gradually increase their charge (stage I), followed by stabilization at an intermediate value associated with MgOH* species (stage II; the asterisk (*) denotes an adsorbed species). During this stage, near-surface Mg atoms are hydroxylated, whereas atoms deeper in the substrate remain metallic. As OH* species migrate further inward (stage III), Mg undergoes deeper hydroxylation, forming amorphous Mg­(OH)_2_^*^, which can subsequently reorganize into crystalline Mg­(OH)_2_. Under conditions favoring the formation of a protective surface film, this pathway dominates.

The second pathway corresponds to the dissolution of hydrated Mg^2+^ ions into the aqueous phase (Figure 7f). While the initial hydroxylation stages are similar to those of the solid-state pathway, Mg atoms located closer to the surface rapidly reach a divalent charge state (stage II, labeled Mg­(OH)_2_^*^ in Figure 7f). These Mg species then detach from the surface, leaving hydroxyl groups behind on the substrate, and form solvated Mg^2+^ ions in solution.

The qNEP model explicitly captures the competing mechanisms of solid-state oxidation and ionic dissolution, highlighting its ability to describe complex electrochemical interfaces with evolving charge states. Overall, the application of qNEP to the Mg–water system not only reproduces key corrosion mechanisms identified in previous studies but also provides dynamic, atom-resolved charge information that reveals the coupled evolution of valence states and structure during magnesium corrosion. This example demonstrates that qNEP overcomes key limitations of traditional MLIPs and enables efficient, large-scale simulations of charge-transfer-driven processes, such as corrosion and electrocatalysis.

## 4 Summary and Conclusions

We have introduced qNEP, a charge-aware extension of the highly efficient NEP framework that incorporates explicit long-range electrostatics while retaining the computational performance required for large-scale MD simulations. In qNEP, partial charges are learned as latent model features without relying on reference charge partitioning, charge conservation is enforced through a dedicated regularization term and a final total-charge correction, and polarization and BECs follow consistently as derivatives of the learned charges. By combining this formulation with an efficient PPPM implementation for reciprocal-space electrostatics, qNEP attains a computational cost only about 1.5–3 times higher than comparably trained NEP models, enabling simulations that extend to million-atom systems and nanosecond-to-tens-of-nanoseconds time scales on consumer-grade GPUs per day. Models that include the short-ranged electrostatic contribution explicitly (mode 1) and those that include only the long-ranged contribution (mode 2) yield similar accuracy.

Across representative liquid, ionic, ferroelectric, and reactive-interface systems, qNEP systematically improves the accuracy of energies, forces, and stresses relative to NEP while providing direct access to charge- and field-related observables. For water, qNEP delivers improved errors at modest overhead and enables infrared spectroscopy via the time ACF of the ionic electric current. For garnet-type Li_7_La_3_Zr_2_O_12_, qNEP captures the tetragonal–cubic transition and reveals phase-dependent charge distributions that correlate with the change in the ionic transport barriers. For BaTiO_3_, qNEP reproduces the sequence of ferroelectric phase transitions, enables predictions of polarization dynamics as well as dielectric response, and natively captures LO–TO in dynamic simulations. For magnesium corrosion in aqueous media, qNEP resolves environment-dependent charge states at a reactive solid–liquid interface and captures competing pathways of solid-state hydroxide formation and dissolution into aqueous Mg^2+^. Together, these results establish qNEP as a practical and scalable route to accurate atomistic simulations of charge-transfer- and polarization-driven phenomena, opening the door to predictive studies of, e.g., transport, dielectric response, spectroscopy, and electrochemical reactivity across extended length and time scales.

Beyond the observables discussed above—including the coupling to external electric fields, dielectric response, and infrared spectroscopy—the results highlight a further, more fundamental advantage of the qNEP approach over simply increasing the cutoff of a short-range model. While larger cutoffs can in principle capture some mid-range interactions, certain effects are intrinsically beyond the reach of any finite-cutoff potential. A paradigmatic example is the LO–TO splitting in polar materials, which arises from a macroscopic electric field and is present in the qNEP results without any post hoc correction. From a practical standpoint, increasing the radial cutoff is also less attractive than it may appear: the computational cost scales as *r*_c_^3^ through the neighbor count, so extending the cutoff from 6 to 8 Å already increases the cost by a factor of approximately 2.4, comparable to the overhead of qNEP itself. Furthermore, larger cutoffs substantially increase the dimensionality of the descriptor space, making models harder to train and often reducing data efficiency.^79^ The qNEP approach therefore provides a more principled, more efficient, and physically more transparent route to long-range interactions in comparison to enlarging the cutoff of a short-range model.

Finally, we note that the present work focuses exclusively on electrostatic interactions as the long-range contribution. Extensions of the qNEP framework to incorporate other long-range interactions, such as dispersion forces,^80–82^ constitute a natural and promising direction for future research. Such developments would further broaden the applicability of the NEP framework, in particular, for aqueous, molecular, and biomolecular systems, where weak electronic screening in water renders electrostatic and other long-range interactions essential.

## Supplementary Material



## Data Availability

The NEP and qNEP models as well as the reference data used for their training and validation have been deposited on Zenodo under the Accession Code 10.5281/zenodo.18335946.

## Notes

Additional information concerning the construction of the MLIP models and the generation of reference data as well as accompanying figures (PDF)
