# Supplementary material for: qNEP: A Highly Efficient Neuroevolution Potential with Dynamic Charges for Large-Scale Atomistic Simulations
Source: J Chem Theory Comput. 2026 Apr 20;22(9):4787–801. doi: 10.1021/acs.jctc.6c00146 (PMC13173505; doi:10.1021/acs.jctc.6c00146)
Supplement: Supplementary file 1 [file ct6c00146_si_001.pdf]

Supporting Information:

qNEP: A highly efficient neuroevolution potential with dynamic charges for large-scale atomistic simulations

Zheyong Fan,[1,2,*] Benrui Tang,[1] Esmée Berger,[3] Ethan Berger,[3] Erik Fransson,[3] Ke Xu,[1] Zihan Yan,[4] Zhoulin Liu,[5] Zichen Song,[6,7] Haikuan Dong,[1] Shunda Chen,[8] Lei Li,[6] Ziliang Wang,[9] Yizhou Zhu,[4] Julia Wiktor,[3] and Paul Erhart[3,10,†]

[1] College of Physical Science and Technology, Bohai University, Jinzhou, P. R. China
[2] Suzhou Laboratory, Suzhou, Jiangsu 215123, P. R. China
[3] Chalmers University of Technology, Department of Physics, 41296 Gothenburg, Sweden
[4] Department of Materials Science and Engineering, Westlake University, Hangzhou, Zhejiang 310030, China
[5] School of Science, Harbin Institute of Technology, Shenzhen 518055, Guangdong, P. R. China
[6] Shenzhen Key Laboratory of Micro/Nano-Porous Functional Materials (SKLPM), Department of Materials Science and Engineering, Southern University of Science and Technology, Shenzhen 518055, China
[7] Department of Materials Science and Engineering, City University of Hong Kong, Hong Kong SAR, China
[8] Department of Civil and Environmental Engineering, George Washington University, Washington, DC 20052, USA
[9] National Engineering Laboratory for Reducing Emissions from Coal Combustion, Shandong Key Laboratory of Green Thermal Power and Carbon Reduction, Shandong University, Jinan, Shandong, P. R. China
[10] Wallenberg Initiative Materials Science for Sustainability, Chalmers University of Technology, 41926 Gothenburg, Sweden
[*] brucenju@gmail.com
[†] erhart@chalmers.se

Contents

Supplementary Notes **S2**
- SN1. General tools . . . S2
- SN2. General note on training . . . S2
- SN3. Training of water models . . . S2
- SN4. DFT calculations for water . . . S2
- SN5. Simulations of water . . . S2
- SN6. Training of lithium lanthanum zirconate $Li_7La_3Zr_2O_{12}$ (LLZO) models . . . S3
- SN7. Simulations of LLZO . . . S3
- SN8. Training of barium titanate models . . . S3
- SN9. DFT calculations for barium titanate . . . S3
- SN10. Simulations of barium titanate . . . S3
- SN11. Phonons in barium titanate . . . S4
- SN12. Training of Mg–O–H models . . . S4
- SN13. Simulations of Mg-water interface . . . S4

Supplementary Tables **S5**
- S1. Comparison of approaches for incorporating long-range electrostatics into MLIPs . . . S5

Supplementary Figures **S6**
- S1. Parity plots for water models . . . S6
- S2. BECs according to qNEP models for water . . . S6
- S3. Convergence of components of the BEC tensor with plane-wave energy cutoff . . . S7
- S4. Speed and production per day on different GPUs . . . S7
- S5. Ratio of the computational speed of NEP vs qNEP models . . . S7
- S6. Parity plots for LLZO models . . . S8
- S7. Charge distribution of Li in LLZO as a function of temperature . . . S8
- S8. Charge distribution of Li in LLZO after relaxation as a function of temperature . . . S9
- S9. Parity plots for $BaTiO_3$ models . . . S9
- S10. BECs according to qNEP models for $BaTiO_3$ . . . S10
- S11. Convergence of BECs for $BaTiO_3$ . . . S10
- S12. Parity plots for Mg–O–H models . . . S10

Supplementary Notes

Supplementary Note 1: General tools

The GPUMD package was used for training neuroevolution potential (NEP) and qNEP models as well as for running all molecular dynamics (MD) simulations (1). The CALORINE package was used to prepare model training and analyze models, as well as to analyze the results of the MD simulations (2). The NEP and qNEP models constructed in this work as well as the reference data used for their training and validation have been deposited on Zenodo under accession code 10.5281/zenodo.18335947.

Supplementary Note 2: General note on training

The $\mathcal{L}_1$ and $\mathcal{L}_2$ regularization weights that enter the NEP or qNEP loss function are set to $\lambda_1 = 0.001/\sqrt{N_{\mathrm{par}}}$ and $\lambda_2 = 0.001$ by default, where N_{par} is the number of trainable parameters per species. The $1/\sqrt{N_{\mathrm{par}}}$ prefactor in λ_1 ensures that the effective regularization strength is independent of model size, accounting for the different scaling of the $\mathcal{L}_1$ norm ($\sim N_{\mathrm{par}}$) and the $\mathcal{L}_2$ norm ($\sim \sqrt{N_{\mathrm{par}}}$) with the number of parameters. All models in this work were trained using these default values.

Supplementary Note 3: Training of water models

We trained NEP and qNEP models in both mode 1 and 2 using the separable natural evolution strategy (SNES) algorithm (3). The radial and angular cutoffs were set to 6 Å and 4 Å, respectively, the descriptor was expressed using $n_{\max} = (4, 4)$ and $l_{\max} = (4, 2)$, and the dimension of the hidden layer was set to 30. We employed a two stage process, in which we first trained the models for 150×10^3 SNES generations emphasizing the contribution of the forces to the loss term ($\lambda_f = 10$, $\lambda_e = 1$, $\lambda_v = 0.1$) followed by another 150×10^3 generations during which the weight of the forces was reduced and the weight of the energies was increased ($\lambda_f = 5$, $\lambda_e = 5$, $\lambda_v = 0.1$). This procedure led to higher accuracies for both energies and forces compared to a single stage optimization of the same total length. For the qNEP models we additionally set the weight of the charge neutrality condition ($\lambda_q = 0.5$) and the Born effective charges (BECs) ($\lambda_{\mathcal{Z}} = 3$), using the same values for both stages. For $\lambda_{\mathcal{Z}}$ we tested values of 1, 3, and 5. Increasing this value reduces the error of the BEC prediction (Figure S2) but if the value becomes too large it negatively affects the other predictions, specifically the forces. The final value of $\lambda_{\mathcal{Z}}$ balances the accuracy of the forces and the BECs.

Supplementary Note 4: DFT calculations for water

The energies, forces, and virials for the reference structures were taken from earlier work (4, 5). For 194 of these structures, we calculated BECs. For consistency with the existing reference data, we employed the strongly constrained and appropriately normed semilocal density functional (SCAN) functional (6) as implemented in the Vienna ab-initio simulation package (7) using projector-augmented wave (8, 9) setups. We tested plane wave energy cutoffs up to 1000 eV and found that 800 eV is sufficient to converge the components of the BEC tensor to below 10^{-3} e (Figure S3). The Brillouin zone was sampled with Γ-centered automatically generated **k**-point grids with a maximum spacing of 0.5/Å.

Supplementary Note 5: Simulations of water

All production runs were carried out using a system comprising 13 000 H_2O molecules at the experimental density at 0.1 MPa (=1 bar) and the respective temperature (10). For reference, at 300 K the density is 996.56 kg/m^3 and it drops to 967.40 kg/m^3.

All systems were equilibrated for 200 ps in the NVT ensemble using a time step of 0.5 fs.

The radial partial radial distribution functions in the classical limit were extracted from the MD trajectory of the equilibrated systems. To incorporate quantum effects path-integral molecular dynamics (PIMD) simulations were carried out (11). In these simulations we used a time step of 0.5 fs and 64 beads. The simulations were run for 200 ps and the partial radial distribution function were sampled every 5 ps.

For the calculation of the infrared spectra, the dynamics were sampled for 400 ps using a time step of 0.1 fs. During the latter part the polarization was evaluated every 0.5 fs using both the BECs and the TNEP model

for the polarization from Ref. 12. The autocorrelation functions (ACFs) of the ionic electric current from the BECs and the polarization from the TNEP model were evaluated and analyzed using the DYNASOR package to obtain the infrared spectra. To improve statistics we ran five simulations for each temperature.

Supplementary Note 6: Training of LLZO models

We trained NEP and qNEP models in both mode 1 and mode 2 using the SNES algorithm (3). To this end, we used the data set of Yan and Zhu (13), which comprises 1978 configurations of pristine LLZO with energies, forces, and stresses computed using the PBEsol exchange–correlation functional (14).

The radial and angular cutoffs were set to 5 Å each, the descriptor was expressed using $n_{\max} = (4, 4)$ and $l_{\max} = (4, 2, 1)$, and the dimension of the hidden layer was set to 30. The universal Ziegler-Biersack-Littmark (ZBL) potential with an outer cutoff of 2 Å and an inner cutoff of 1 Å was applied to improve the robustness of the machine-learned interatomic potential (MLIP). Models were trained for 100×10^3 SNES generations using $\lambda_f = 1$, $\lambda_e = 1$, $\lambda_v = 0.1$, and $\lambda_q = 0.1$.

Supplementary Note 7: Simulations of LLZO

The heating and cooling simulations were carried out in the NPT ensemble using a $6 \times 6 \times 6$ supercell (41 472 atoms) and a time step of 2 fs. The temperature was varied between 200 K and 1200 K over a period of 20 ns. The heat capacity was obtained by taking the numerical derivative of the potential energy after application of a gliding average.

The MD simulations for the ion diffusion utilized a $4\times4\times4$ supercell (12 288 atoms) and Martyna-Tuckerman-Tobias-Klein integrators (15) for sampling the NPT ensemble. The tetragonal and cubic phases were simulated for 2 ns and 1 ns, respectively, using a timestep of 1 fs. The Li diffusivity was calculated from the mean square displacement over time,

$$D = \frac{1}{2Nd\Delta t} \sum_{i=1}^{N} \left\langle |\mathbf{r}_i(t + \Delta t) - \mathbf{r}_i(t)|^2 \right\rangle_t , \tag{1}$$

where N is the total number of diffusing ions, $d = 3$ denotes the dimension of the system, $r_i(t)$ is the displacement of ion i at time t, and the bracket represents averaging over t. The ionic conductivity (σ) is then determined using the Nernst-Einstein relation

$$\sigma = \frac{nq^2}{k_{\mathrm{B}}T} D, \tag{2}$$

where n is the number of mobile ions per unit volume, q is the ionic charge, k_B is the Boltzmann constant, and T is the temperature.

Supplementary Note 8: Training of barium titanate models

An initial set of 639 structures was taken from Ref. 16 (see `https://doi.org/10.5281/zenodo.15283532` for the Zenodo record). This set comprises fully relaxed bulk structures covering various phases including rhombohedral, orthorhombic, tetragonal, cubic, and hexagonal variants across a wide range of volumes. It also features rattled structures, structures with controlled displacements along the ferroelectric modes, as well as structures from two generations of active learning. This set was augmented by adding structures with BECs. These were obtained by running MD simulations using the NEP model previously trained in Ref. 16. In total, 1193 structures of $\sqrt{2} \times \sqrt{2} \times 2$ supercells (20 atoms) were obtained at temperatures between 50 K and 600 K to sample all four phases. The final reference set comprised 1832 structures, corresponding to a total of 36 540 atoms.

The radial and angular cutoffs were set to 6 Å and 4 Å, respectively, the descriptor was expressed using $n_{\max} = (8, 6)$ and $l_{\max} = (4, 0)$, and the dimension of the hidden layer was set to 40. We trained NEP and qNEP models in both mode 1 and mode 2 using the SNES algorithm (3). Models were trained for 500×10^3 SNES generations using $\lambda_f = 1$, $\lambda_e = 1$, $\lambda_v = 0.1$, $\lambda_q = 0.1$, and $\lambda_{\mathcal{Z}} = 0.1$.

Supplementary Note 9: DFT calculations for barium titanate

The energies, forces, and virials for the initial 639 reference structures were taken from earlier work (16). For consistency, energies, forces and virials for the additional 1193 structures are obtained using the r2SCAN exchange-correlation functional (17) as implemented in the Vienna ab-initio simulation package (7). The BECs were then obtained using density functional perturbation theory and the PBEsol functional (14).

Supplementary Note 10: Simulations of barium titanate

First, cooling simulations were performed in the NPT ensemble using the qNEP model and starting from a perfectly cubic $20 \times 20 \times 20$ supercell (40 000 atoms). A time step of 1 fs was used for all simulations. After 100 ps of equilibration at 500 K, the temperature was linearly decreased to 50 K over 45 ns, resulting in a cooling rate of 10 K/ns. The final structure was then used as the starting point for a heating run with the same heating rate of 10 K/ns.

For both cooling and heating simulations, 100 structures were saved along the trajectory and subsequently used to obtain the lattice parameters, the spontaneous polarization and the dielectric function as a function of temperature. The spontaneous polarization $\mathbf{P}$ was obtained as

$$P_\alpha = \frac{1}{V} \sum_{i=1}^{N} \sum_{\beta} \mathcal{Z}_{i\alpha\beta} u_{i\beta}, \tag{3}$$

where V is the volume and $\mathbf{u}_i = \mathbf{r}_i - \mathbf{r}_0$ denotes the displacement of atom i from its reference position $\mathbf{r}_0$ in the cubic structure. The dielectric constants were obtained as

$$\varepsilon_{\alpha\beta} = 4\pi \frac{P_\alpha(E \cdot \hat{e}_\beta) - P_\alpha(0)}{E}, \tag{4}$$

where $P_\alpha(\mathbf{E})$ denotes the polarization in the Cartesian direction α when applying an external electric field $\mathbf{E}$. In practice, we ran MD simulations both without and with electric field in each Cartesian direction, and computed the average polarization as $P_\alpha(\mathbf{E})$. The strength of the electric field was set to 1 mV/Å.

Polarization–electric field (P–E) hysteresis loops were obtained by running MD simulations at constant temperature but with the field varying from 0.2 V/nm to −0.2 V/nm over 2 ns (backward) and again from −0.2 V/nm to 0.2 V/nm over 2 ns (forward), corresponding to a frequency of 500 MHz. The atomic positions were sampled every 20 ps and used to obtain the polarization via Eq. (3).

The imaginary part of the dielectric function was obtained from the Fourier transform of the time ACF $\langle \dot{\mathbf{P}}(0) \cdot \dot{\mathbf{P}}(t) \rangle$. The time derivative of the polarization $\dot{\mathbf{P}}(t)$ was sampled every 20 fs along a 2 ns MD trajectory at constant temperature and volume, and calculated using Eq. (32) of the main text. The real part of the dielectric function was subsequently obtained via a Kramer-Kronig transformation.

Supplementary Note 11: Phonons in barium titanate

The harmonic phonons were evaluated using PHONOPY in supercells (320 atoms) of $4 \times 4 \times 4$ repetitions of the cubic primitive cell (18, 19). The phonon spectral energy density (20) was evaluated from MD simulations in the NVE ensemble at 500 K in the cubic phase using supercells of $36 \times 36 \times 36$ repetitions (233 280 atoms). The simulations were run using a time step of 1 fs, using lattice parameters from the NPT runs. The system was first equilibrated for 100 ps in the NVT ensemble, followed by 150 ps in the NVE ensemble where velocities were saved to file every 15 fs. The spectral energy density was then computed from the velocities using DYNASOR (21, 22).

Supplementary Note 12: Training of Mg–O–H models

The reference dataset contained 2809 structures, obtained from Ref. 23, for which the energies, forces, and stresses were calculated using density-functional theory (DFT) and the PBE exchange–correlation functional (24). This dataset includes metallic magnesium, aqueous solutions, hydrogen gas, magnesium oxides, magnesium hydrides, magnesium hydroxides, and interfacial structures between magnesium metal and water. This dataset was augmented with D3 dispersion corrections for training.

The radial and angular cutoffs were set to 6.5 Å and 4.5 Å, respectively, the descriptor was expressed using $n_{\max} = (4, 4)$ and $l_{\max} = (4, 2)$, and the dimension of the hidden layer was set to 30. We trained a NEP model and a qNEP model in mode 2 using the SNES algorithm (3). Models were trained for 100×10^3 SNES generations using $\lambda_f = 1$, $\lambda_e = 1$, $\lambda_v = 0.1$, and $\lambda_q = 0.1$.

Supplementary Note 13: Simulations of Mg-water interface

A comprehensive analysis of the corrosion of Mg in water was recently presented in Ref. 23 based on a (charge unaware) NEP model. Since the detailed trajectories are determined by a series of rare events, a comprehensive understanding of this system requires a large number of simulations and analysis. Here, we are focused on the development of the qNEP approach. Rather than repeating the comprehensive analysis from Ref. 23 we therefore applied our qNEP model to analyze a specific trajectory from this earlier work.

The MD simulation shown in the main text was taken from Ref. 23 and generated using the NEP model developed in the same work. While the potential was fitted without DFT-D3 (PBE) dispersion corrections, the latter were applied during the MD run to improve the description of the interactions with the water environment. The qNEP model (Note 12) was used to analyze the evolution of the charge distribution.

The simulation shown contains approximately 2430 atoms and a highly reactive stepped surface of magnesium, the construction of which is described in Ref. 25. To ensure a sufficiently reactive environment, the H_2O:Mg ratio was chosen to be greater than two. The system was first relaxed in the NPT ensemble at 300 K and 1 bar, followed by a production run in the NVT ensemble at 700 K. The system was simulated for 5 ns using a time step of 0.5 fs.

Supplementary Tables

Table S1: Comparison of selected approaches for incorporating long-range electrostatics into interatomic potentials. "Explicit" charges are fitted to reference values from a charge-decomposition scheme; "latent" charges are not fitted to reference values but emerge from fitting the total energy. "BEC/P" indicates whether BECs and the polarization are directly available as physically consistent response properties. Computational overhead is relative to an equivalently trained short-range MLIP of the same architecture.

Approach	Charge type	Charge conservation	BEC/P	Repr. refs.
Fixed-charge subtraction	Fixed	Exact	No	26
3G-HDNNP (regression charges)	Explicit	Via correction	No	27, 28
Dipole/Wannier-function	Implicit	By construction	Yes (P)	29–31
4G-HDNNP (charge equilibration)	Self-consistent	Exact	No	32–34
Electric enthalpy / equivariant NN	Latent	N/A	Yes	35
Latent charges (full Ewald)	Latent	Via correction	Yes	36
Latent charges (k-space only Ewald)	Latent	Via optional correction	Yes	37–40
qNEP (particle-particle particle-mesh)	Latent	Via regularization + correction	Yes	This work

Supplementary Figures

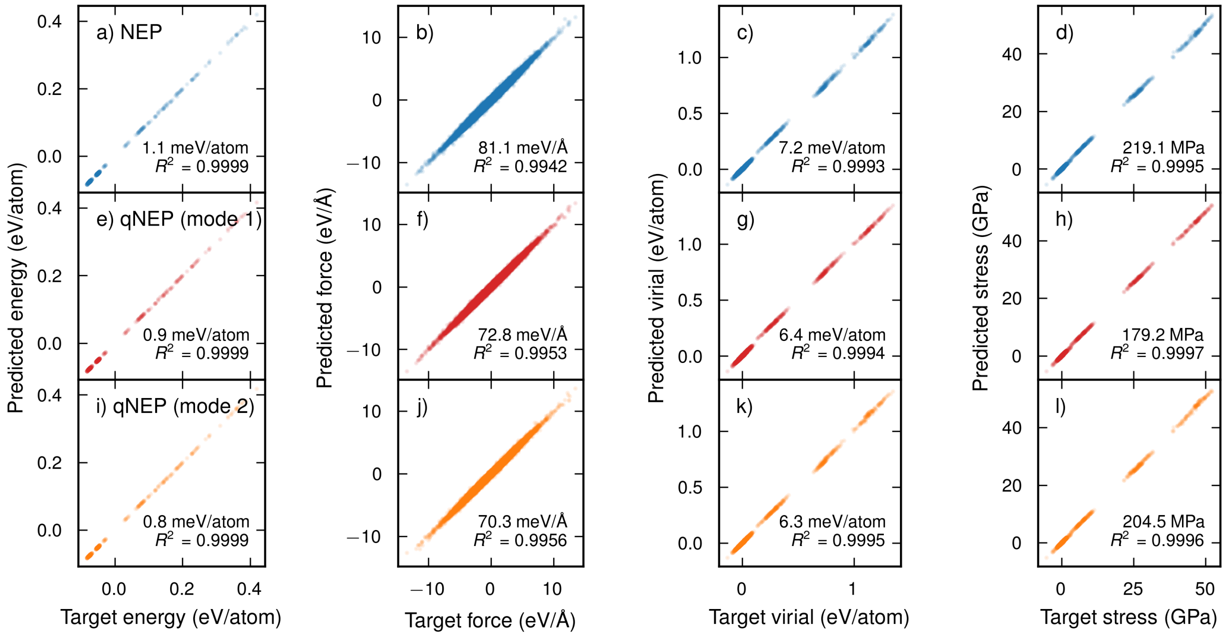

Figure S1: Energy, forces, virials, and stresses of water configurations (validation set) for NEP and qNEP models compared to target data from DFT calculations.

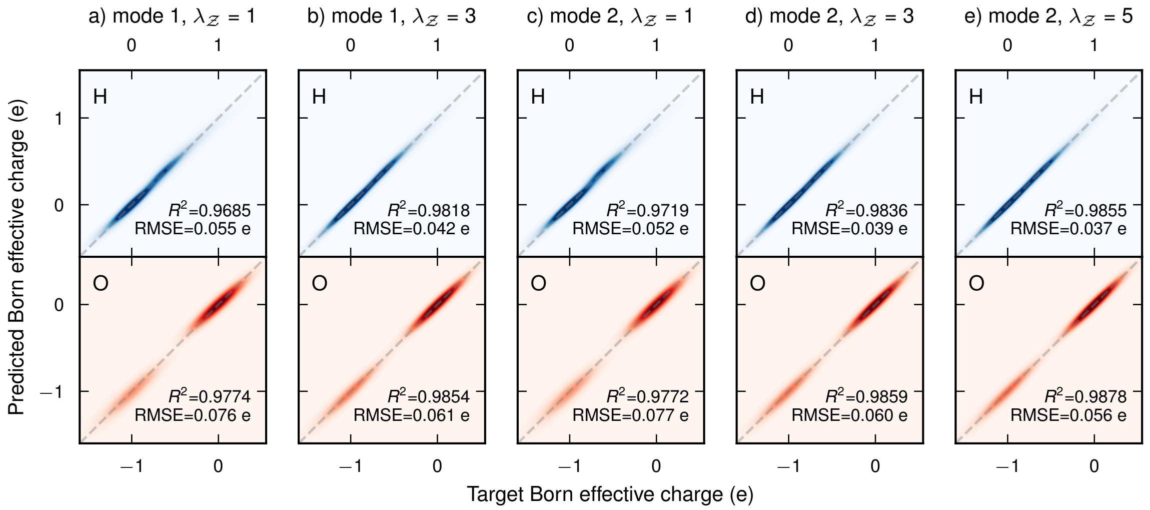

Figure S2: Kernel density estimate plots of BECs in water predicted by qNEP models trained using mode 1 and mode 2 as well as different choices for the $\lambda_{\mathcal{Z}}$ parameter compared to target data from DFT calculations. The latter controls the weight of the BECs in the loss function. The insets provide the respective coefficients of correlation (R^2) and root-mean-square errors (RMSEs).

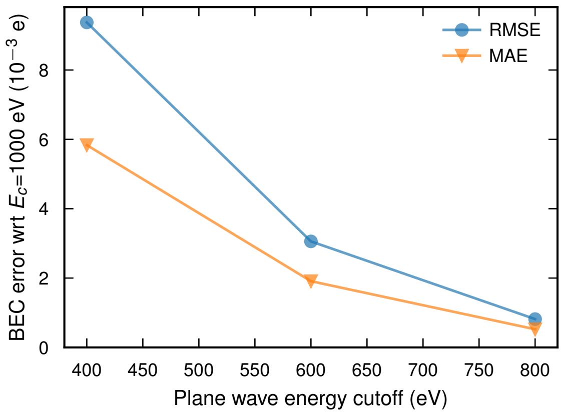

Figure S3: Convergence of components of the BEC tensor in water with plane-wave energy cutoff.

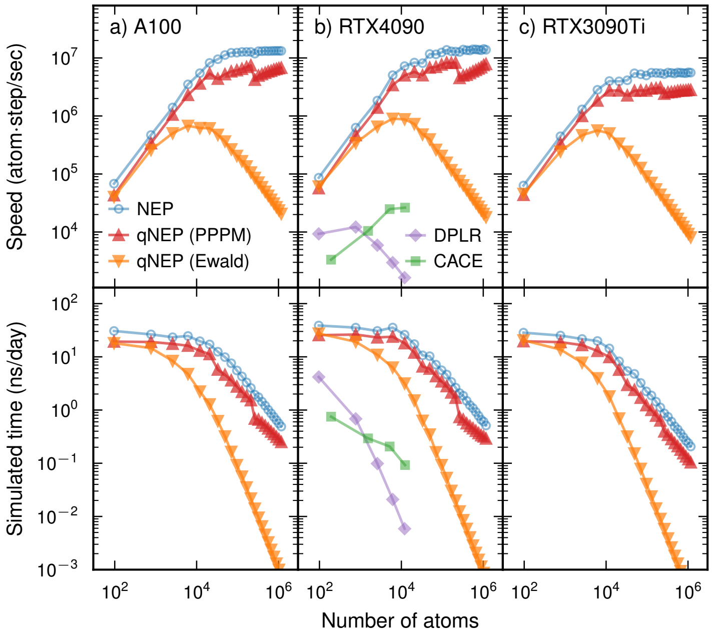

Figure S4: Computational speed (top) and corresponding simulated time (bottom) for NEP and qNEP models for water on different GPUs. Simulations were carried out at 300 K and a density of 1000 kg/m^3 using a time step of 0.5 fs.

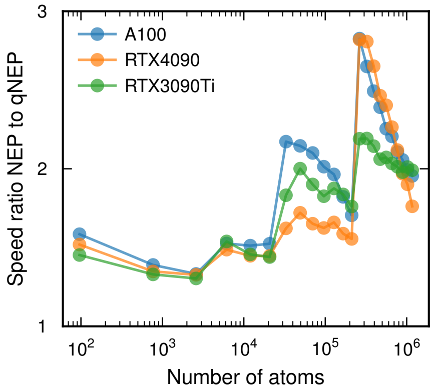

Figure S5: Ratio of the computational speed of NEP and qNEP models (mode 2) for water on different GPUs. Simulations were carried out at 300 K and a density of 1000 kg/m^3.

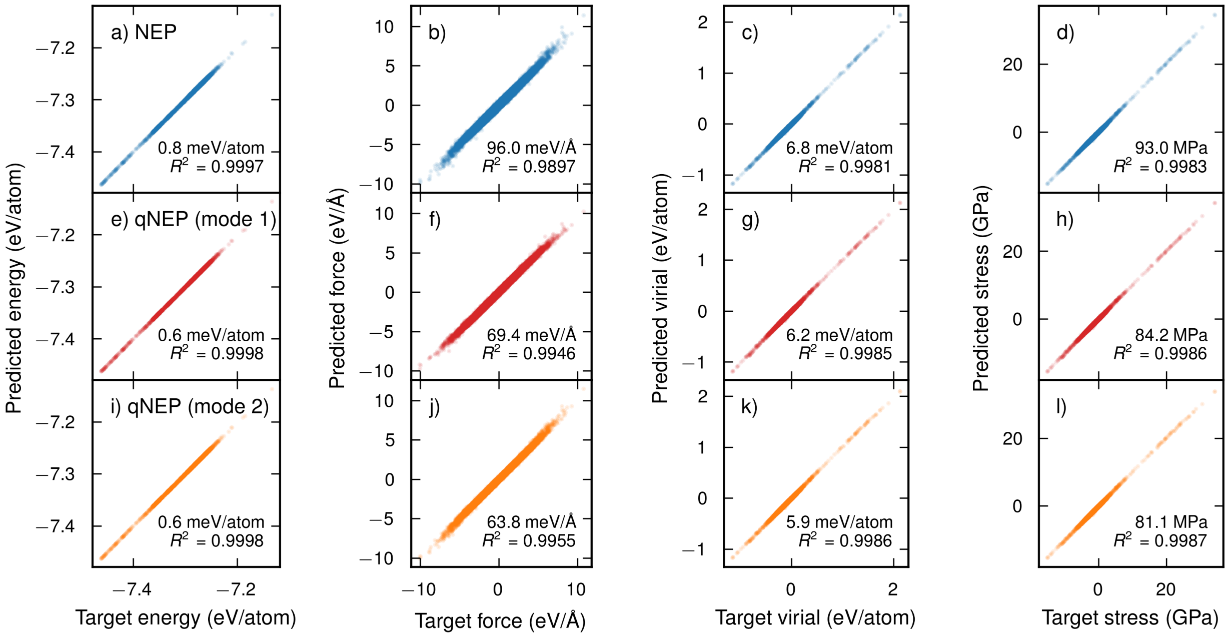

Figure S6: Parity plots for energy, forces, virials, and stresses for the reference configurations for LLZO for NEP and qNEP models compared to target data from DFT calculations.

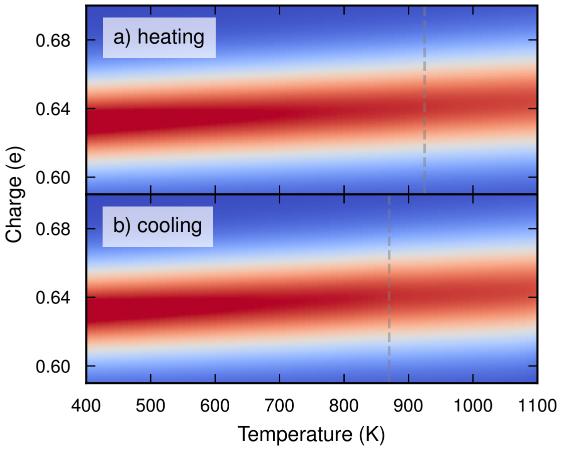

Figure S7: Charge distribution of Li as a function of temperature LLZO from kernel density estimates from (a) heating and (b) cooling runs.

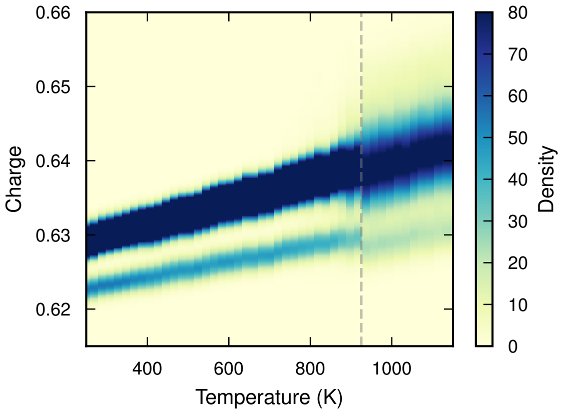

Figure S8: Charge distribution of Li in LLZO as a function of temperature from kernel density estimates. The snapshots were obtained at different temperatures along a heating run and subsequently relaxed with the cell metric fixed. Note the abrupt change in the charge distribution at the transition from the low-temperature t-LLZO to the high-temperature c-LLZO phase.

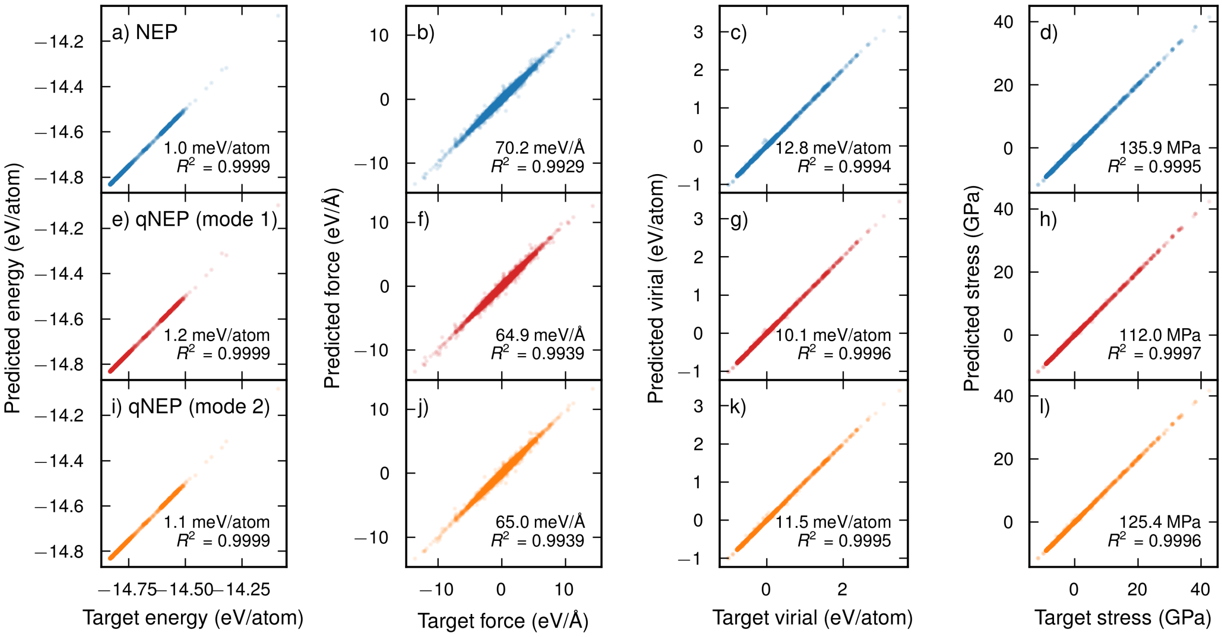

Figure S9: Parity plots for energy, forces, virials, and stresses for the reference configurations for $BaTiO_3$ for NEP and qNEP models compared to target data from DFT calculations.

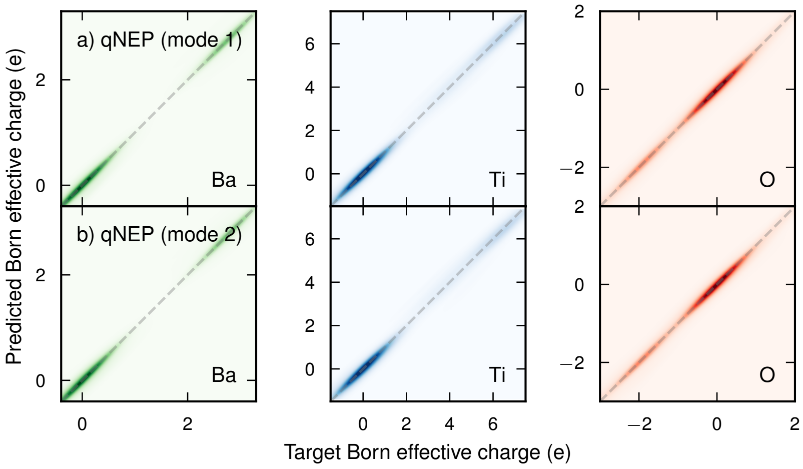

Figure S10: Kernel density estimate plots of BECs in $BaTiO_3$ predicted by qNEP models trained using (a) mode 1 and (b) mode 2 in comparison with target data from DFT calculations.

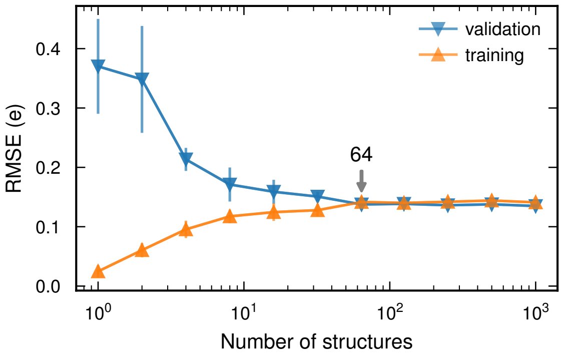

Figure S11: Convergence of the predictive accuracy for BECs in $BaTiO_3$ from qNEP models trained using a variable number of structures with BEC information. In this case the RMSE is converged with as few as 64 structures, illustrating that only relatively few structures with BECs are needed for training.

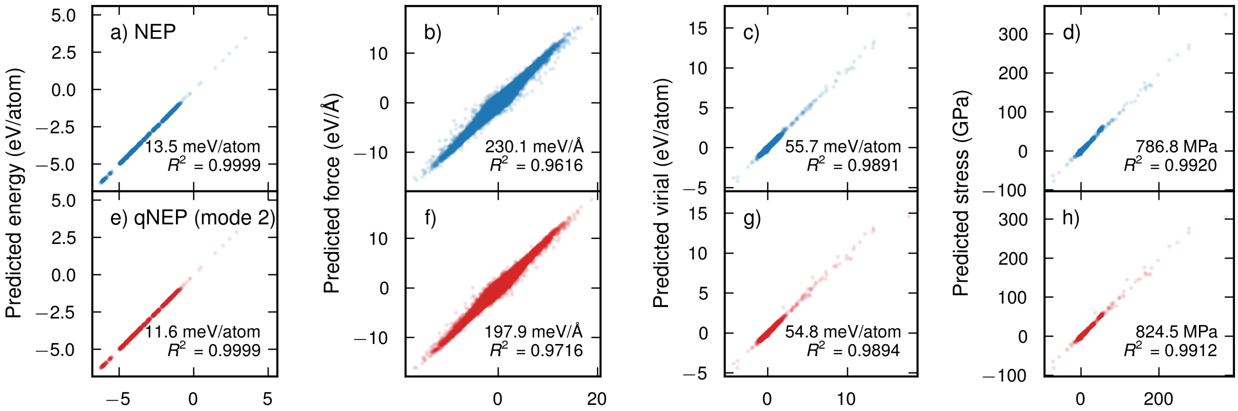

Figure S12: Parity plots for energy, forces, virials, and stresses for the reference configurations for the Mg–O–H system from NEP and qNEP models, where the latter has been trained using mode 2, compared to target data obtained from DFT calculations.

Supplementary References

[1] K. Xu, H. Bu, S. Pan, E. Lindgren, Y. Wu, Y. Wang, J. Liu, K. Song, B. Xu, Y. Li, T. Hainer, L. Svensson, J. Wiktor, R. Zhao, H. Huang, C. Qian, S. Zhang, Z. Zeng, B. Zhang, B. Tang, Y. Xiao, Z. Yan, J. Shi, Z. Liang, J. Wang, T. Liang, S. Cao, Y. Wang, P. Ying, N. Xu, C. Chen, Y. Zhang, Z. Chen, X. Wu, W. Jiang, E. Berger, Y. Li, S. Chen, A. J. Gabourie, H. Dong, S. Xiong, N. Wei, Y. Chen, J. Xu, F. Ding, Z. Sun, T. Ala-Nissila, A. Harju, J. Zheng, P. Guan, P. Erhart, J. Sun, W. Ouyang, Y. Su, and Z. Fan, *GPUMD 4.0: A high-performance molecular dynamics package for versatile materials simulations with machine-learned potentials,* Materials Genome Engineering Advances **3**, e70028 (2025). `doi:doi:10.1002/mgea.70028`.

[2] E. Lindgren, M. Rahm, E. Fransson, F. Eriksson, N. Österbacka, Z. Fan, and P. Erhart, *Calorine: A Python package for constructing and sampling neuroevolution potential models,* Journal of Open Source Software **9**, 6264 (2024). `doi:10.21105/joss.06264`.

[3] T. Schaul, T. Glasmachers, and J. Schmidhuber, *High dimensions and heavy tails for natural evolution strategies,* in *Proceedings of the 13th Annual Conference on Genetic and Evolutionary Computation*, GECCO '11, (New York, NY, USA), 845852, Association for Computing Machinery, 2011. `doi:10.1145/2001576.2001692`.

[4] L. Zhang, H. Wang, R. Car, and W. E, *Phase diagram of a deep potential water model,* Physical Review Letters **126**, 236001 (2021). `doi:10.1103/PhysRevLett.126.236001`.

[5] K. Xu, Y. Hao, T. Liang, P. Ying, J. Xu, J. Wu, and Z. Fan, *Accurate prediction of heat conductivity of water by a neuroevolution potential,* The Journal of Chemical Physics **158**, 204114 (2023). `doi:10.1063/5.0147039`.

[6] J. Sun, A. Ruzsinszky, and J. P. Perdew, *Strongly constrained and appropriately normed semilocal density functional,* Physical Review Letters **115**, 036402 (2015). `doi:10.1103/PhysRevLett.115.036402`.

[7] G. Kresse and J. Furthmüller, *Efficiency of ab-initio total energy calculations for metals and semiconductors using a plane-wave basis set,* Computational Materials Science **6**, 15 (1996). `doi:10.1016/0927-0256(96)00008-0`.

[8] P. E. Blöchl, *Projector augmented-wave method,* Physical Review B **50**, 17953 (1994). `doi:10.1103/PhysRevB.50.17953`.

[9] G. Kresse and D. Joubert, *From ultrasoft pseudopotentials to the projector augmented-wave method,* Physical Review B **59**, 1758 (1999). `doi:10.1103/PhysRevB.59.1758`.

[10] E. W. Lemmon, I. H. Bell, M. L. Huber, and M. O. McLinden, *Thermophysical Properties of Fluid Systems,* in *NIST Chemistry WebBook, NIST Standard Reference Database Number 69*, edited by P. J. Linstrom and W. G. Mallard (Gaithersburg MD, 20899: National Institute of Standards and Technology, retrieved January 11, 2026). `doi:10.18434/T4D303`.

[11] P. Ying, W. Zhou, L. Svensson, E. Berger, E. Fransson, F. Eriksson, K. Xu, T. Liang, J. Xu, B. Song, S. Chen, P. Erhart, and Z. Fan, *Highly efficient path-integral molecular dynamics simulations with GPUMD using neuroevolution potentials: Case studies on thermal properties of materials,* Journal of Chemical Physics **162**, 064109 (2025). `doi:10.1063/5.0241006`.

[12] N. Xu, P. Rosander, C. Schäfer, E. Lindgren, N. Österbacka, M. Fang, W. Chen, Y. He, Z. Fan, and P. Erhart, *Tensorial properties via the neuroevolution potential framework: Fast simulation of infrared and Raman spectra,* Journal of Chemical Theory and Computation **20**, 3273 (2024). `doi:10.1021/acs.jctc.3c01343`.

[13] Z. Yan and Y. Zhu, *Impact of lithium nonstoichiometry on ionic diffusion in tetragonal garnet-type $Li_7La_3Zr_2O_{12}$,* Chemistry of Materials **36**, 11551 (2024). `doi:10.1021/acs.chemmater.4c02454`.

[14] J. P. Perdew, A. Ruzsinszky, G. I. Csonka, O. A. Vydrov, G. E. Scuseria, L. A. Constantin, X. Zhou, and K. Burke, *Restoring the density-gradient expansion for exchange in solids and surfaces,* Physical Review Letters **100**, 136406 (2008). `doi:10.1103/PhysRevLett.100.136406`.

[15] G. J. Martyna, M. E. Tuckerman, D. J. Tobias, and M. L. Klein, *Explicit reversible integrators for extended systems dynamics,* Molecular Physics **87**, 1117 (1996). `doi:10.1080/00268979600100761`.

[16] E. Lindgren, A. J. Jackson, E. Fransson, E. Berger, S. Rudić, G. Škoro, R. Turanyi, S. Mukhopadhyay, and P. Erhart, *Predicting neutron experiments from first principles: A workflow powered by machine learning,* Journal of Materials Chemistry A **13**, 25509 (2025). doi:10.1039/D5TA03325J.

[17] J. W. Furness, A. D. Kaplan, J. Ning, J. P. Perdew, and J. Sun, *Accurate and numerically efficient r2SCAN meta-generalized gradient approximation,* The Journal of Physical Chemistry Letters **11**, 8208 (2020). doi:10.1021/acs.jpclett.0c02405.

[18] A. Togo, L. Chaput, T. Tadano, and I. Tanaka, *Implementation strategies in phonopy and phono3py,* Journal of Physics: Condensed Matter **35**, 353001 (2023). doi:10.1088/1361-648X/acd831.

[19] A. Togo, *First-principles phonon calculations with Phonopy and Phono3py,* Journal of the Physical Society of Japan **92**, 012001 (2023). doi:10.7566/JPSJ.92.012001.

[20] J. A. Thomas, J. E. Turney, R. M. Iutzi, C. H. Amon, and A. J. H. McGaughey, *Predicting phonon dispersion relations and lifetimes from the spectral energy density,* Physical Review B **81**, 081411 (2010). doi:10.1103/PhysRevB.81.081411.

[21] E. Fransson, M. Slabanja, P. Erhart, and G. Wahnström, *dynasor–A tool for extracting dynamical structure factors and current correlation functions from molecular dynamics simulations,* Advanced Theory and Simulations **4**, 2000240 (2021). doi:10.1002/adts.202000240.

[22] E. Berger, E. Fransson, F. Eriksson, E. Lindgren, G. Wahnström, T. H. Rod, and P. Erhart, *Dynasor 2: From simulation to experiment through correlation functions,* Computer Physics Communications **316**, 109759 (2025). doi:10.1016/j.cpc.2025.109759.

[23] Z. Liu, J. Sha, G.-L. Song, Z. Wang, and Y. Zhang, *Understanding magnesium dissolution through Machine learning molecular dynamics,* Chemical Engineering Journal **516**, 163578 (2025). doi:10.1016/j.cej.2025.163578.

[24] J. P. Perdew, K. Burke, and M. Ernzerhof, *Generalized gradient approximation made simple,* Physical Review Letters **77**, 3865 (1996). doi:10.1103/PhysRevLett.77.3865.

[25] Z. Liu, J. Bao, J. Sha, and Z. Zhang, *Modulation of the discharge and corrosion properties of aqueous Mg–air batteries by alloying from first-principles theory,* The Journal of Physical Chemistry C **127**, 10062 (2023). doi:10.1021/acs.jpcc.3c00111.

[26] Z. Deng, C. Chen, X.-G. Li, and S. P. Ong, *An electrostatic spectral neighbor analysis potential for lithium nitride,* npj Computational Materials **5**, 75 (2019). doi:10.1038/s41524-019-0212-1.

[27] N. Artrith, T. Morawietz, and J. Behler, *High-dimensional neural-network potentials for multicomponent systems: Applications to zinc oxide,* Physical Review B **83**, 153101 (2011). doi:10.1103/PhysRevB.83.153101.

[28] T. Morawietz, V. Sharma, and J. Behler, *A neural network potential-energy surface for the water dimer based on environment-dependent atomic energies and charges,* The Journal of Chemical Physics **136**, 064103 (2012). doi:10.1063/1.3682557.

[29] O. T. Unke and M. Meuwly, *PhysNet: A Neural Network for Predicting Energies, Forces, Dipole Moments, and Partial Charges,* Journal of Chemical Theory and Computation **15**, 3678 (2019). doi:10.1021/acs.jctc.9b00181.

[30] L. Zhang, H. Wang, M. C. Muniz, A. Z. Panagiotopoulos, R. Car, and W. E, *A deep potential model with long-range electrostatic interactions,* The Journal of Chemical Physics **156**, 124107 (2022). doi:10.1063/5.0083669.

[31] A. Gao and R. C. Remsing, *Self-consistent determination of long-range electrostatics in neural network potentials,* Nature Communications **13**, 1572 (2022). doi:10.1038/s41467-022-29243-2.

[32] T. W. Ko, J. A. Finkler, S. Goedecker, and J. Behler, *A fourth-generation high-dimensional neural network potential with accurate electrostatics including non-local charge transfer,* Nature Communications **12**, 398 (2021). doi:10.1038/s41467-020-20427-2.

[33] T. W. Ko, J. A. Finkler, S. Goedecker, and J. Behler, *Accurate Fourth-Generation Machine Learning Potentials by Electrostatic Embedding,* Journal of Chemical Theory and Computation **19**, 3567 (2023). doi:10.1021/acs.jctc.2c01146.

[34] M. Gubler, J. A. Finkler, M. R. Schäfer, J. Behler, and S. Goedecker, *Accelerating Fourth-Generation Machine Learning Potentials Using Quasi-Linear Scaling Particle Mesh Charge Equilibration,* Journal of Chemical Theory and Computation **20**, 7264 (2024). `doi:10.1021/acs.jctc.4c00334`.

[35] S. Falletta, A. Cepellotti, A. Johansson, C. W. Tan, M. L. Descoteaux, A. Musaelian, C. J. Owen, and B. Kozinsky, *Unified differentiable learning of electric response,* Nature Communications **16**, 4031 (2025). `doi:10.1038/s41467-025-59304-1`.

[36] Z. Song, J. Han, G. Henkelman, and L. Li, *Charge-Optimized Electrostatic Interaction Atom-Centered Neural Network Algorithm,* Journal of Chemical Theory and Computation **20**, 2088 (2024). `doi:10.1021/acs.jctc.3c01254`.

[37] B. Cheng, *Latent Ewald summation for machine learning of long-range interactions,* npj Computational Materials **11**, 80 (2025). `doi:10.1038/s41524-025-01577-7`.

[38] D. S. King, D. Kim, P. Zhong, and B. Cheng, *Machine learning of charges and long-range interactions from energies and forces,* Nature Communications **16**, 8763 (2025). `doi:10.1038/s41467-025-63852-x`.

[39] P. Zhong, D. Kim, D. S. King, and B. Cheng, *Machine learning interatomic potential can infer electrical response,* npj Computational Matererials **11**, 384 (2025). `doi:10.1038/s41524-025-01911-z`.

[40] D. Kim, X. Wang, S. Vargas, P. Zhong, D. S. King, T. J. Inizan, and B. Cheng, *A Universal Augmentation Framework for Long-Range Electrostatics in Machine Learning Interatomic Potentials,* Journal of Chemical Theory and Computation **21**, 12709 (2025). `doi:10.1021/acs.jctc.5c01400`.
